# Molecular Chain Elongation Mechanism for *n*‐Caproate Biosynthesis by *Megasphaera Hexanoica*

**DOI:** 10.1002/advs.202506069

**Published:** 2025-09-11

**Authors:** Byoung Seung Jeon, Eun‐Jung Kim, Hogyun Seo, Hyunjin Kim, Seungjin Shin, Caroline Schlaiß, Largus T. Angenent, Kyung‐Jin Kim, Byoung‐In Sang

**Affiliations:** ^1^ Korea Institute of Ceramic Engineering and Technology Osong 28160 Republic of Korea; ^2^ School of Life Sciences KNU Creative BioResearch Group KNU Institute for Microorganisms Kyungpook National University Daegu 41566 Republic of Korea; ^3^ Viral Disease Division Animal and Plant Quarantine Agency 177, Hyeoksin 8‐ro Gimcheon‐si Gyeongbuk 39660 Republic of Korea; ^4^ Department of Chemical Engineering Hanyang University Seoul 04763 Republic of Korea; ^5^ AG Angenent Max Planck Institute for Biology Max Planck Ring 5 D‐72076 Tübingen Germany; ^6^ Department of Biological and Chemical Engineering Aarhus University Gustav Wieds vej 10D Aarhus C 8000 Denmark; ^7^ Environmental Biotechnology Group Department of Geosciences University of Tübingen Schnarrenbergstraße 94–96 72076 Tübingen Germany; ^8^ The Novo Nordisk Foundation CO2 Research Center (CORC) Aarhus University Gustav Wieds vej 10C Aarhus C 8000 Denmark; ^9^ Cluster of Excellence – Controlling Microbes to Fight Infections University of Tübingen Auf der Morgenstelle 28 72074 Tübingen Germany

**Keywords:** β‐Ketothiolase, Chain elongation mechanism, Medium‐chain carboxylates, *Megasphaera hexanoica*, *n*‐Caproate biosynthesis, Protein structure analysis, Site‐directed mutagenesis

## Abstract

The microbial production of medium‐chain carboxylates has attracted considerable interest owing to their potential applications in biofuels and specialty chemicals; however, the underlying biosynthetic mechanisms remain incompletely understood. The present study evaluates the medium‐chain carboxylate‐producing microbe *Megaspahera hexanoica* using genomic analysis, transcriptome analysis, and metabolic engineering. Additionally, the *n*‐caproate synthesis pathway of *M. hexanoica* is characterized with fructose as an electron donor, and the substrate specificity of the respective proteins is evaluated by constructing an *n*‐caproate biosynthetic pathway in *Escherichia coli*. Among all r‐BOX or RBO genes, *thl*_1583, which encodes β‐ketothiolase (*Mh*THL), is identified as the most critical enzyme for the carbon chain elongation mechanism in *M. hexanoica*. Therefore, *Mh*THL is compared with other well‐studied β‐ketothiolases (*Ck*THL from *Clostridium kluyveri, Re*BktB from *Ralstonia eutropha* (*Cupriavidus necator*), *Ec*AtoB from *E. coli*, and *Ca*THL from *C. acetobutylicum*). *Mh*THL is found to exhibit the highest *n*‐caproate production, as evidenced by the protein crystal structure of *Mh*THL. Structural comparisons with other thiolases show that *Mh*THL has a larger substrate‐binding pocket than *Re*BktB. Thiolase mutants generated by site‐directed mutagenesis reveal that two residues (Leu87 and Val351) are essential for determining the size of the substrate‐binding pocket.

## Graphical abstract

The medium‐chain carboxylate‐producing microbe *Megasphaera hexanoica* is analyzed through genomics, transcriptomics, and metabolic engineering. The *n*‐caproate biosynthetic pathway is reconstructed in *Escherichia coli*, highlighting *Mh*THL as a key β‐ketothiolase. Structural analysis and mutagenesis reveal that *Mh*THL has a distinct substrate‐binding pocket, crucial for *n*‐caproate production.

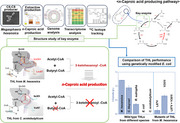

## 1 Introduction

Medium‐chain carboxylates (MCCs) are aliphatic straight carbon chains of 6–12 carbon atoms widely used in fine chemical applications, such as artificial flavors, rubber chemicals, varnish driers, resins, plasticizers, and pharmaceuticals.^[^ ^1^ ^]^ Production of MCCs has gained particular interest in the past decade owing to their valorization potential via secondary chemical reactions.^[^ ^2^ ^]^ MCCs are conventionally produced from an aldehyde or a longer‐chain fatty acid via oxidation, typically utilizing fossil‐based or plant‐derived carbon sources.^[^ ^3^ ^]^ However, microbial chain elongation using renewable resources has been suggested as an alternative for achieving sustainability.^[^ ^4^ ^]^ Microbial production is advantageous as it recovers resources from waste and uses equipment, such as anaerobic digesters, without significant modification.^[^ ^5^ ^]^ Thus, bio‐derived MCCs can potentially serve fine chemical and transport fuel markets in the near future. The MCC *n*‐caproate (*n*‐hexanoate; C_6_) contains one carboxylic group and can be converted into longer‐chain hydrocarbons via Kolbe Electrolysis, with the potential to produce drop‐in fuel.^[^ ^6^ ^]^

Well‐known bacterial strains that produce *n*‐caproate include *Megasphaera elsdenii*, *Caproiciproducens * spp., and *Clostridium kluyveri*, which are often observed in chain elongation bioprocesses using open cultures of microbial consortia. These bacteria are thought to produce *n‐*caproate via the reverse β‐oxidation (r‐BOX, also known as RBO) pathway.^[^ ^7^ ^]^ However, given that their genomes do not provide information for distinguishing between *n*‐butyrate (C_4_), *n*‐caproate, and *n*‐caprylate synthesis, it is unclear how they produce medium‐chain carboxylic acids, such as *n*‐caproate or *n*‐caprylate (C_8_). Consequently, the phylogenetic classification of r‐BOX genes from *n*‐caproate producers is similar to that of genes involved in *n*‐butyrate metabolism (e.g., *Clostridium acetobutylicum* and *C. tyrobutyricum*, which do not produce carboxylic acids longer than C_4_).^[^ ^8^ ^]^ It is, therefore, challenging to pinpoint at the genomic level why and how *n*‐caproate is produced. Elucidating the differences between *n*‐butyrate and *n*‐caproate production via the r‐BOX pathway at the genomic level is expected to provide insights into chain elongation. Although *n*‐butyrate and *n*‐caproate are both synthesized via the reverse β‐oxidation (r‐BOX) pathway, their high pathway similarity has made mechanistic differentiation difficult. Genomic‐level comparison may help identify key enzymes specific to *n*‐caproate production, offering new targets for improving chain elongation efficiency.

We previously evaluated the MCC production performance of *M. hexanoica*, revealing high *n*‐caproate production and prominent characteristics of chain elongation mechanisms, producing MCCs of various lengths from exogenously added short‐chain carboxylates (SCCs, *i.e*., carboxylic acids between C_2_ and C_4_ such as acetate, propionate, and *n*‐butyrate).^[^ ^9^ ^]^ In the current study, production of *n*‐caproate by *M. hexanoica* was assessed at the genomic, transcriptome, metabolic, and protein structure levels. Core genes related to *n*‐caproate production were selected to establish the *n*‐caproate pathway of *M. hexanoica*. The findings of the present study could enhance our understanding of the mechanisms of biological chain elongation, thereby enhancing the utility of bio‐derived compounds and fostering the development of the biorefinery industry.

## 2 Results

### 2.1 Elucidating the Mechanism of Carbon Chain Elongation in *M. hexanoica*

**Figure 1:**
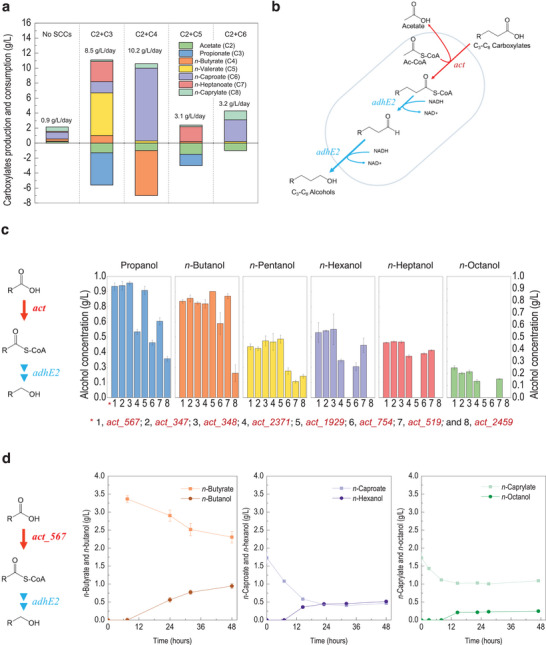
The reverse β‐oxidation (rBOX) pathway, with acetate CoA‐transferases, is responsible for SCC uptake and MCC production. a) Fermentation products and productivity according to various electron acceptors by *M. hexanoica* using fructose. b) Alcohol‐production pathway involving acetate CoA‐transferases. c) Screening ACTs in *M. hexanoica* using engineered *E. coli*. d) Time course of carboxylate consumption and the corresponding alcohol production using engineered *E. coli* with *act_*567. Abbreviations: *act*, acyl‐CoA transferase; *thl*, thiolase; *hbd*, 3‐hydroxy butyryl‐CoA dehydrogenase; *crt*, crotonase; *acdh*, acyl‐CoA dehydrogenase; *etf* αβ, electron transfer protein subunit A and subunit B; and *adhE2*, aldehyde‐alcohol dehydrogenase. Figure 1a was derived from the mean values reported in Table S1 (Supporting Information), with each mean calculated from two technical replicates per sample using Microsoft Excel. To enhance clarity, standard deviations are not displayed in Figure 1a; however, the corresponding mean ± standard deviation (SD) values are provided in Table S1 (Supporting Information). The results in Figure 1c,d are presented as mean ± SD, calculated from two technical replicates per sample using Origin software.

*Megasphaera hexanoica* selectively produces MCCs using sugars as electron donors.^[^ ^10^ ^]^ *Megasphaera hexanoica* formed odd‐numbered carboxylic acids, such as *n*‐valerate (C_5_) and *n*‐heptanoate (C_7_), when propionate (C_3_) was added (**Figure** **1**a; Figure S1, and Table S1, Supporting Information).^[^ ^9e^ ^]^ Likewise, supplementation with even‐numbered carboxylates (e.g., acetate and *n*‐butyrate) induced *n*‐caproate production (Figure 1a). When *n‐*butyrate was supplemented with fructose (0.1 m each), *n*‐caproate production increased to 6.34 g L^−1^ (Table S1, Supporting Information). Interestingly, with acetate and *n*‐butyrate supplementation, *n*‐caproate concentration ultimately increased to 10.63 g L^−1^, achieving 0.43 g L h^−1^ productivity (Figures S2 and S3, Supporting Information). As well, *n*‐butyrate was preferentially consumed (6.3 g L^−1^), while acetate appeared to be partially consumed (1.3 g L^−1^), resulting in its persistence at a relatively stable concentration. To further clarify the specific contribution of acetate to the *n*‐caproate synthesis pathway, we compared its effect in various substrate combinations (Table S1, Supporting Information). When acetate was co‐supplemented with fructose (0.1 m each), *n*‐caproate production increased markedly from 0.88 g L^−1^ (fructose only) to 4.37 g L^−1^, demonstrating its effective role as an electron acceptor during chain elongation. In addition, the co‐supplementation of acetate tended to increase cell growth, which suggests that acetate facilitates intracellular redox balance through the contribution of the r‐BOX metabolic pathway. As a result, these effects have led to increased production of *n*‐caproate. Surprisingly, when *n‐*caproate was added externally, *M. hexanoica* elongated the supplemented MCCs by two additional carbons to *n*‐caprylate (C_8_), reaching a final concentration of 1.15 g L^−1^. Compared to *Clostridium kluyveri*, which typically produces *n*‐caprylate at concentrations of 0.3–0.5 g L^−1^ under optimized conditions,^[^ ^11^ ^]^ *M. hexanoica* demonstrates high C_8_ production efficiency in a pure culture system (Figure 1a; Table S1, Supporting Information). Thus, *M. hexanoica* can produce MCCs from externally added carboxylates and fructose.

The produced MCCs were further evaluated by adding ^13^C‐labeled *n*‐butyrate and unlabeled fructose; the MCC molecular structures were determined via mass spectrometry. ^13^C‐labeled *n*‐butyrate was incorporated into *n*‐caproate, while ^13^C‐labeled isotopes were at positions 3, 4, 5, and 6 of the *n*‐caproate carbon, starting at the carboxylic group (Figure S4, Supporting Information). The first and second carbons of *n*‐caproate were carbon‐12 (^12^C), derived from the unlabeled electron donor fructose. Hence, *n*‐caproate production in *M. hexanoica* follows chain elongation rules by adding two carbons simultaneously from position 1 in the carboxylate.

To confirm the continuous elongation of the two carbon atoms, *M. hexanoica* was cultivated in a fed‐batch reactor equipped with an in situ extractive fermentation system for 23 days (Figure S5, Supporting Information). An increase in pH was observed in the fermentation broth because of the continuous extraction of undissociated *n*‐caproate from the biphasic system (Figure S5, Supporting Information). The biphasic system consisted of a 0.7 L fermentation broth and a 1.4 L extraction solvent composed of a 9:1 mixture of oleyl alcohol and Alamine 336. During fermentation, undissociated *n*‐caproate was continuously extracted into the solvent layer, resulting in a gradual increase in the pH of the fermentation broth. To counteract this, *n*‐butyrate was added as a buffering electron acceptor whenever the pH rose, maintaining the pH within the optimal range of 5.9–6.0 using a pH auxostat. Fructose (electron donor) and concentrated nutrient solution were spike‐fed when the fructose concentration dropped below 5 g L^−1^. Under these conditions, *n*‐caproate of 182 g was produced, with a maximum volumetric production rate of 0.27 g L h^−1^ (Figure S5 and Table S4, Supporting Information). The production yield achieved was relatively high, reaching 0.92 moles of CA per mole of carbon source. As shown in the results using ^13^C butyrate, *n*‐butyrate seemed to be employed in the synthesis of *n*‐caproate. As a result, the continuous *n*‐butyrate consumption led to a progressive increase in the selectivity for *n‐*caproate. When the selectivity of *n*‐caproate was calculated from total carboxylic acids, it was ultimately reached at 89.14%. However, the intermittent spike feeding approach caused noticeable fluctuations in optical density (OD_600nm_) over time, suggesting that the sudden addition of concentrated fructose and nutrients may have imposed osmotic or metabolic stress on the cells, thereby reducing productivity. To overcome this limitation, the feeding strategy was switched to a semi‐fed‐batch mode, where concentrated fructose and nutrients were continuously supplied using a syringe pump (Figure S6, Supporting Information). In the semi‐fed‐batch mode, the volumetric production rate significantly improved, reaching up to 2.0 g L h^−1^, and a total of 70 g of *n*‐caproate was produced within two days, which represents the highest reported productivity achieved by a pure culture system (Figure S6 and Table S4, Supporting Information). As shown in the fed‐batch mode, the semi‐fed batch also achieved a considerably high yield of 0.86 mole CA /mole carbon source. These results demonstrate the feasibility of developing a cost‐effective biotechnological platform for *n*‐caproate production using *M. hexanoica* under optimized fermentation and feeding conditions.

### 2.2 Determination of the *n*‐Caproate Biosynthetic Pathway in *M. hexanoica* using Transcriptome Analysis and Genetically Engineered *E. coli*

For *n*‐caproate production via the r‐BOX pathway, conversion of *n*‐butyrate to butyryl‐coenzyme A (butyryl‐CoA) is essential. Thus, potentially corresponding enzymes of *M. hexanoica* were screened after gene annotation using the Basic Local Alignment Search tool. Eight acetate CoA‐transferases (ACT, EC 2.8.3.8) capable of converting SCCs to short‐chain acyl‐CoA were detected in the *M. hexanoica* genome (Figure S7 and Table S5, Supporting Information), double the number in the genome of its closest relative (i.e., *Megasphaera elsdenii*) (Figure S7 and Table S5, Supporting Information). Transcriptome results revealed that *act*_567 and *act*_347 showed relatively higher differential expression than other *act* genes (Figure S8a, Supporting Information). Both genes are similar in terms of enzymatic function and phylogenetic classification. Thus, these enzymes were predicted to participate in the transfer of CoA molecules between the MCCs and acyl‐CoA.

ACTs convert carboxylates into acyl‐CoAs, and the *C. acetobutylicum* alcohol dehydrogenase (AdhE2) produces alcohols from acyl‐CoAs. Therefore, the substrate specificity of the *act* genes was evaluated by constructing a linear metabolic pathway in *E. coli* involving two enzymes that can transform acyl‐CoA molecules into their respective alcohols (Figure 1b; Figure S9 and Tables S7 and S8, Supporting Information). The *act* genes were inserted into the empty multiple cloning site of the duet vector plasmid bearing the *adhE2* gene, which is related to *n*‐butanol production. The carboxylates were converted into acyl‐CoA molecules via ACT activity; AdhE2 subsequently transformed acyl‐CoA into alcohols, such as *n*‐butanol and *n‐*hexanol. Additionally, *act*_567, *act*_347, and *act*_348 performed better than other ACTs with *n*‐caproate, *n*‐heptanoate, and *n‐*caprylate (Figure 1c,d). Similar to the results of previous studies,^[^ ^12^ ^]^ *act*_567 exhibited the highest performance, even in terms of *n*‐octanol production. Accordingly, *act*_567 was selected to construct an *n*‐caproate biosynthetic pathway in *E. coli*.

In addition to ACTs, transcriptome results showed that genes related to the r‐BOX pathway were highly expressed (Figures S8b and S10, and Table S8, Supporting Information). These were genes transcribing thiolase (THL), 3‐hydroxybutyryl‐CoA dehydrogenase (HBD), enoyl‐CoA hydratase/crotonase (CRT), butyryl‐CoA dehydrogenase Etf complex (BCDH/Etf αβ), and acyl‐CoA dehydrogenase (ACDH), which are also essential enzymes of the r‐BOX pathway. *n*‐Caproate producers, such as *M. elsdenii* and *C. kluyveri*, have more than two thiolases that perform the condensation reaction of two acetyl‐CoA molecules, whereas only one thiolase (*thl*_1583) was observed in the genome of *M. hexanoica* (Figure S7 and Table S5, Supporting Information). Transcription of *thl*_1583 showed high expression within the third‐highest expression ranks during the *n*‐caproate‐producing exponential phase (AB9, e.g., 9 h, exponential phase) (Figure S10a and Table S8, Supporting Information). The expression level of *thl*_1583 was higher than that of the other genes, even under non‐*n*‐caproate producing conditions (N9, e.g., 9 h, exponential phase, and N19, e.g., 19 h, stationary phase). However, the expression of *thl*_1583 appeared was significantly reduced after 18 h under *n*‐caproate‐producing conditions, leading to a decrease in *n*‐caproate production (Figure S3, Supporting Information). The overall expression pattern in AB18 also differed from that in the other conditions (AB8, N9, and N19). Clearly, *n*‐caproate accumulated in the culture broth under condition AB18, and this accumulated *n*‐caproate affected cell growth and metabolite production (Figure S3, Supporting Information). Therefore, we sought to elucidate the relationship between thiolase expression and *n*‐caproate production.

The *M. hexanoica* genome contained two pairs: *hbd*, *crt*, *crt*_613, *hbd*_2207, *crt*_2206, and *hbd*_2534. *hbd*_2207 and *crt*_2206 were adjacent to each other (Figure S7 and Table S5, Supporting Information). Distinctively, the transcription of *hbd*_2207 and *crt*_2206 exhibited the second‐ and eighth‐highest expression levels during the exponential phase (AB9) of *n*‐caproate production (Figure S8a and Table S8, Supporting Information). In contrast, *crt*_613 and *hbd*_2534 maintained low expression levels under all conditions (Figure S8b, Supporting Information). Unfortunately, *acdh* could not be specified exclusively as the four *acdh* genes exhibited high similarity. These genes were located at different positions in the genome, with codon sequence numbers *acdh*_56, *acdh*_612, *acdh*_2230, and *acdh*_2251 (Figure S7 and Table S5, Supporting Information). *acdh*_2230 is closely related to BCDHs and is referred to as *bcdh*_2230. In particular, *acdh*_2251 and *bcdh*_2230 exhibited the sixth‐ and 15th‐highest expression in the transcriptome of the *n‐*caproate‐producing exponential phase (AB9), respectively (Figure S8a and Table S8, Supporting Information). Therefore, *act*_567, *thl*_1583, *hbd*_2207, *crt*_2206, *bcdh*_2230, and *acdh*_2251 were selected as the crucial genes in *n*‐caproate production by *M. hexanoica*. In addition, *etf* αβ subunits were included as a coenzyme for *n*‐caproate production.^[^ ^13^ ^]^ Moreover, the trans‐enoyl‐CoA reductase (*ter*) derived from *Treponema denticola* served as a positive control for *n‐*caproate production, as it is widely used for carboxylate production in synthetic biology.^[^ ^14^ ^]^ Generally, *etf* αβ subunits are highly conserved in *n*‐butyrate‐producing anaerobes and are associated with *bcdh*.^[^ ^15^ ^]^ Likewise, the *etf* αβ subunits connected to *bcdh*_2230 were *etf* α_2229 and *etf* β_2228, which were chosen to verify *n*‐caproate production as they were ranked 9th and 12th with significantly higher expression levels under exponential conditions (Figure S8a and Table S8, Supporting Information).

**Figure 2:**
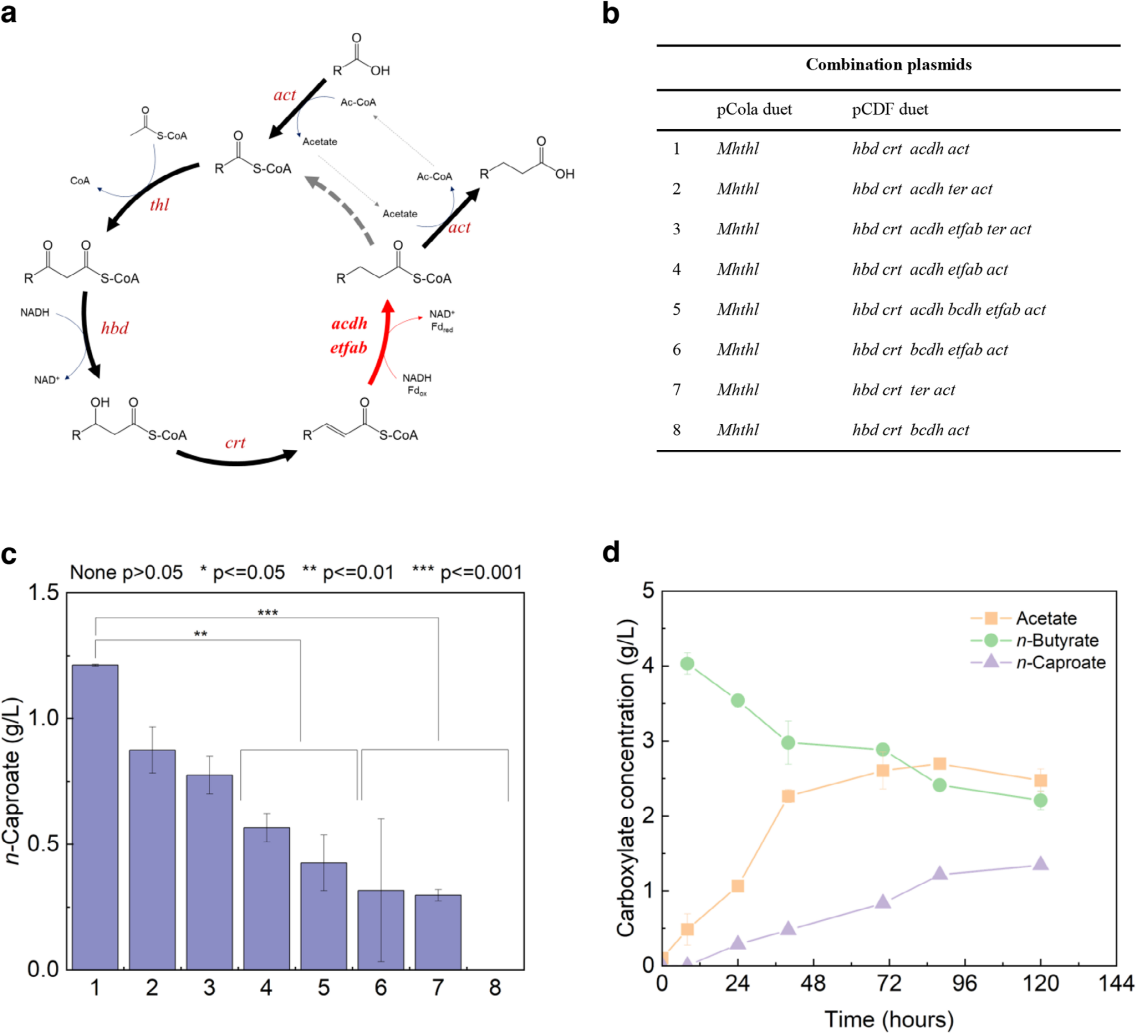
Verification of *n*‐caproate production by r‐BOX genes derived from *M. hexanoica*. a) Putative reverse β‐oxidation pathway of *M. hexanoica*. b) Composition and location of selected genes in vectors pCOLA and pCDF duet, and the list of genes constructed in this study. c) Production of *n*‐caproate using combinatorial plasmids. d) Time course of carboxylate concentrations in culture broth inoculated with the engineered *E. coli* with optimum gene combinations. Abbreviations: *act*, acyl‐CoA transferase; *thl*, thiolase; *hbd*, 3‐hydroxy butyryl‐CoA dehydrogenase; *crt*, crotonase; *acdh*, acyl‐CoA dehydrogenase; *etf*αβ, electron transfer protein subunit A and subunit B; *Mhthl*, thiolase from *M. hexanoica*; and ter, trans‐enoyl‐CoA reductase. All samples in Figure 2c,d were analyzed in technical duplicate by two independent operators under identical experimental conditions. Data are presented as mean ± SD, calculated using Origin software. Statistical differences between groups in Figure 2c were assessed using paired *t*‐tests, with *p* < 0.05 considered statistically significant. Levels of significance are denoted as follows: ^*^ *p* < 0.05, ^**^ *p* < 0.01, ^***^ *p* < 0.001, none, not significant (*p* > 0.05).

Eight *E. coli* strains were genetically engineered (**Figure** **2**b; Figure S12, Supporting Information) to test the candidate genes. Specifically, *bcdh_*2230, *acdh*_2251, *ter*, and *etf* αβ complex were evaluated as these genes exhibited considerable correlations in their functional activity. The *bcdh_*2230, *etf* αβ complex, *acdh*_2251, and *ter* as potential candidates participate in the saturation step (conversion of trans‐2‐hexenoyl‐CoA to hexanoyl‐CoA) of the r‐BOX pathway (Figure 2a; Table S9, Supporting Information). We compared the effects of different enzymes on *n*‐caproate production via the eight strains (Figure S12, Supporting Information). The first plasmid combination with *thl*_1583, *hbd*_2207, *crt*_2206, *acdh*_2251, and *act*_567 led to the highest *n*‐caproate production with a concentration of 1.25 g L^−1^ (Figure 2c,d), despite the absence of *etf* αβ subunits. Meanwhile, *acdh*_2251 was not accompanied by *etf* αβ in the *M. hexanoica* genome (Figure S7, Supporting Information) and exhibited the sixth‐highest transcriptome expression under the *n‐*caproate‐producing conditions (Figure S8 and Table S8, Supporting Information). Similarly, the strain carrying the *ter* gene produced *n*‐caproate, however, at a lower rate than the strain containing *acdh*_2251 (Figure 2c). Combining *bcdh*_2230 with *etf* αβ produced a reduced level with the highest deviation in *n*‐caproate production (Figure 2c). Therefore, we hypothesized that *bcdh*_2230 functions in *n*‐butyrate production rather than *n*‐caproate.^[^ ^15a^ ^]^

The optimal combination of heterologous genes for *n*‐caproate production was the *E. coli* ACT#1, comprising the genes with the highest RNA expression levels in the *M. hexanoica* transcriptome analysis (Figure S8 and Table S8, Supporting Information). Therefore, *thl*_1583, *hbd*_2207, *crt*_2206, *acdh*_2251, and *act*_567 were deemed the main constituents in the *M. hexanoica n*‐caproate biosynthetic pathway. Accordingly, the most promising thiolase of *M. hexanoica* was *thl*_1583 (*Mh*THL), which catalyzes the first reaction in the r‐BOX pathway and is the most promising for *n*‐caproate production in *E. coli*.

### 2.3 *Mh*THL is a Crucial Enzyme in *M. hexanoica n*‐Caproate Production

**Figure 3:**
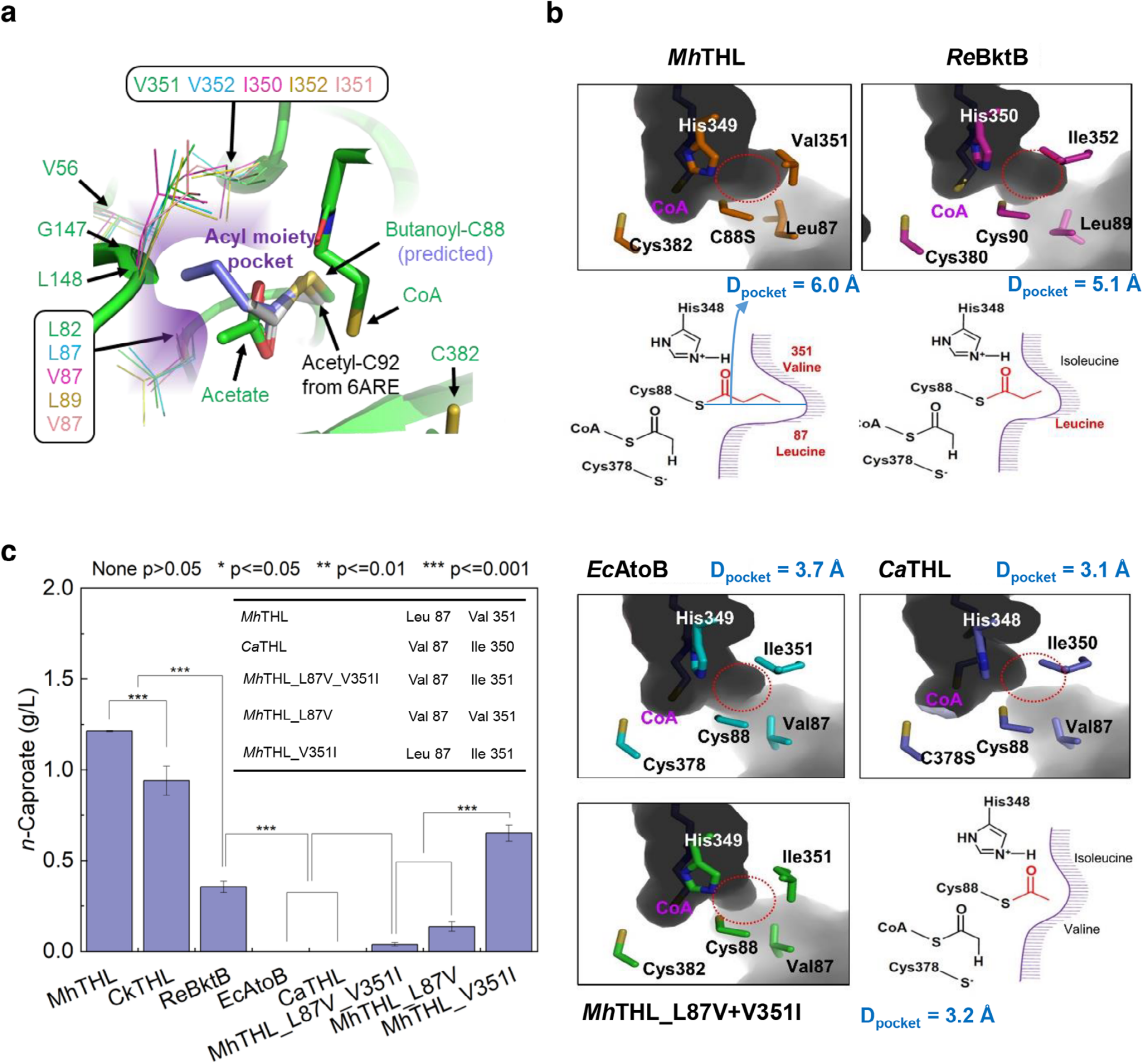
Structural insights for thiolase in *n*‐caproate production. a) Acyl moiety binding pocket of *Mh*THL and key residues. The *Mh*THL structure is shown as a green cartoon model. The bound CoA and acetate molecules are shown with a stick model. C88S residue is replaced by a butanoyl‐C88 residue, and the predicted atoms are distinguished with light blue. The residues constituting the acyl moiety binding pocket in *Mh*THL (Green), *Ec*AtoB (Pink, PDB code: 5F0V), *Re*BktB (Yellow, PDB code 4W61), *Ca*THL (Magenta, PDB code 413), and *Ck*THL (Cyan, PDB code 8JG3) are shown with line models. The key residue positions (L82 and V351) are distinguished in hatched ovals, and the corresponding positions in the other thiolases are labeled. b) Pocket size differences among the thiolases. D_pocket_ is the distance from the sulfur atom of cysteine 88 to the end of the pocket in the direction passing between the two key residues. c) Comparisons of *n*‐caproate production for different thiolases. All samples in Figure 3c were analyzed in technical duplicate by two independent operators under identical experimental conditions. Data are presented as mean ± standard deviation (SD), calculated using Origin software. Statistical differences between groups were assessed using paired t‐tests in Origin, with *p* < 0.05 considered statistically significant. Levels of significance are indicated as follows: *p* < 0.05, *p* < 0.01, *p* < 0.001; n.s., not significant (*p* > 0.05).

Four thiolases from other well‐studied bacteria were selected, and their activities were compared using the same expression platform in *E. coli* (Figure S13, Supporting Information). The THL from *C. kluyveri* (*Ck*THL) was selected as *C. kluyveri* serves as a model *n*‐caproate producer.^[^ ^16^ ^]^ BktB from *Ralstonia eutropha* (*Re*BktB) was also chosen, as it is related to polyhydroxyalkanoate production and has been demonstrated to enable *n*‐hexanol or *n*‐octanol production.^[^ ^14^, ^17^ ^]^ Additionally, the thiolase AtoB derived from *E. coli* (*Ec*AtoB) was included, which has been widely used for *n*‐butyrate or *n*‐butanol production in synthetic biology.^[^ ^18^ ^]^ The thiolase THL from *C. acetobutylicum* (*Ca*THL) was incorporated and is the most feasible strain for *n*‐butanol production.^[^ ^19^ ^]^ The respective thiolase activities were compared by inserting *Ck*THL *Re*BktB, *Ec*AtoB, *Ca*THL, and *Mh*THL into the platform strain *E. coli* SK‐1 (Table S10, Supporting Information). The strain containing *Mh*THL produced more *n*‐caproate than the strains encoding other thiolase genes, reaching a final concentration of 1.25 g L^−1^ (**Figure** **3**c). As expected, the strains containing *Ec*AtoB or *Ca*THL did not produce *n*‐caproate (Figure 3c), indicating that these enzymes did not utilize butyryl‐CoA as an acyl group donor. *Re*BktB treatment produced approximately one‐third (0.36 g L^−1^) of the *n*‐caproate concentration of *Mh*THL (Figure 3c). However, the strain containing *Ck*THL produced a similar level (≈0.9 g L^−1^) of *n*‐caproate as *Mh*THL (Figure 3c). Hence, *Mh*THL showed the best performance for C_6_ elongation among the well‐characterized thiolases. In addition, we postulate that the properties of thiolases determine the carbon length of the end products and that the high *n*‐caproate production of *M. hexanoica* results from its native thiolase.

### 2.4 Structural Features of Thiolase in *n*‐Caproate Production

To determine why the *Mh*THL‐modified *E. coli* strain produced *n*‐caproate, we assessed the structure of *Mh*THL at a 1.64 Å resolution using its C88S variant crystals soaked in a hexanoyl‐coenzyme A (CoA) solution (Figure S14, Supporting Information). The monomeric structure of *Mh*THL comprises three specific domains: 1) N‐domain (residues 1–117 and 252–272), 2) C‐terminal domain (residues 273–393), and 3) loop domain (residues 118–251) (Figures S15b and S5, Supporting Information). *Mh*THL exhibited the typical topology of a type II biosynthetic thiolase fold with a tetramerization motif (residues 123–142) in the loop domain (Figures S14b and S15, Supporting Information). Although the crystals were soaked in hexanoyl‐CoA, a deacylated CoA molecule was bound to *Mh*THL in the m*F*_observed_‐D*F*_calculated_ electron density map (Figure S16, Supporting Information). According to the catalytic mechanism of the enzymatic Claisen condensation,^[^ ^20^ ^]^ the S‐to‐O substituent of the covalent catalyst C88 and the second nucleophile C382 were positioned toward the thiol group of the CoA molecule (Figure S14c, Supporting Information). The experimental X‐ray diffraction data showed a significant electron density for the carboxylate ion between the main‐chain N atoms of C88S and G384. We theorized that the map position in all chains of the asymmetric unit was occupied by a carboxylate (acetate molecule in the model) (Figure S16, Supporting Information), owing to the thioesterase activity of the C‐to‐S variant of thiolase.^[^ ^21^ ^]^ This model might indicate the presence of an oxyanion hole and the approximate position of the caproate moiety of hexanoyl‐CoA.

We then superimposed the *Mh*THL structure on the crystal structures of well‐known thiolases, such as *Ec*AtoB, *Re*BktB, *Ca*THL, and *Ck*THL, to investigate their structural differences. The overall structures were relatively identical, with alpha carbon RMS distances of 0.50, 0.62, 0.53, and 0.46, respectively (Figure S17, Supporting Information). The positioning of the C88S oxygen atom in *Mh*THL differs from that of the sulfur atom of the corresponding cysteine in other thiolases, with a ≈−37° rotation of the Oγ‐Cβ‐Cα‐N dihedral angle (Figure S18, Supporting Information). The sulfur atoms of the other structures collided with the carboxylate ion, further supporting the existence of carboxylate ions in our model (Figure S18, Supporting Information). The acyl moiety binding pocket of *Mh*THL can be predicted using the thioester bond of C88, inferred from the carboxylate ion and tetrahedral intermediate structure of a cytosolic thiolase from *Aspergillus fumigatus* (Figure 3a).^[^ ^22^ ^]^ The predicted pocket in *Mh*THL was composed primarily of hydrophobic residues, such as V56, L87, G147, L148, and V351 (Figure 3a). L87 and V351 in *Mh*THL and the corresponding L87 and V352 in *Ck*THL modulated the pocket size to a greater extent than the valine and isoleucine residues in *Ec*AtoB and *Ca*THL (Figure 3b; Table S11, Supporting Information). In the case of *Re*BktB, the protein contains Leu89 and Ile352 at the positions, making the pocket larger than *Ca*THL but smaller than *Ck*THL and *Mh*THL (Figure 3b; Table S11, Supporting Information). This corresponds to the *n*‐caproate production results in the platform strain SK‐1 with altered thiolases (Figure 3c).

Based on these results, we identified the Leu87 and Val351 residues in *Mh*THL as key residues for utilizing acyl‐CoA molecules as acyl group donors. To elucidate whether these are important for *n*‐caproate production, *Mh*Thl^L87V^, *Mh*Thl^V351I^, and *Mh*Thl^L87V/V351I^ mutant genes were generated (Tables S11 and S12, Supporting Information) and heterologously expressed in *E. coli* SK‐1 cells to compare their *n*‐caproate production performance. All mutants exhibited reduced *n*‐caproate production (Figure 3c), indicating that these residues participated in 3‐ketohexanoyl‐CoA production. Notably, *Mh*THL^L87V/V351I^ showed markedly decreased caproate production (0.04 g L^−1^) compared to that seen with wild‐type *Mh*THL (Figure 3c). Accordingly, we suggest that the Leu87 and Val351 residues in *Mh*THL are key structural features for determining the substrate specificity of biosynthetic thiolases, providing *M. hexanoica* the ability to produce *n*‐caproate.

## 3 Discussion

In the current study, the *n*‐caproate production pathway was established using *M. hexanoica* genes to address questions regarding the chain elongation mechanism. Although *n‐*caproate is typically produced by a metabolic cascade that combines single enzymes, the r‐BOX pathway in *M. hexanoica* appeared more complex than in other wild‐type bacteria.^[^ ^15c^ ^]^ Moreover, the previously defined r‐BOX pathway in *M. hexanoica* was hypothesized to be incomplete because of the absence of thioesterases.

Thioesterases have been reported to play a role in determining the chain length of carboxylates by catalyzing the hydrolysis of acyl‐CoAs in the r‐BOX pathway of wild‐type bacteria^[^ ^23^ ^]^ and genetically modified microbes.^[^ ^14^, ^17^, ^24^ ^]^ Accordingly, we screened putative thioesterases. We detected one acyl‐CoA hydrolase gene (*ach*_2716, 444 bp) in the *M. hexanoica* genome, and its potential for *n*‐caproate liberation was observed through heterologous expression in *E.coli*. Enzymatic activity was assessed using a DTNB‐based colorimetric assay that detects CoA‐SH release from hexanoyl‐CoA. Only negligible activity was observed, suggesting minimal hydrolytic function against hexanoyl‐CoA under the tested conditions. In addition, transcriptomic data revealed that *ach*_2716 was expressed at significantly lower levels than *act*_0567 under *n*‐caproate producing conditions (Figure S8, Supporting Information). Its RNA expression was between 46.02 and 79.58 relative log expression (RLE) under conditions of AB9 and AB18, respectively. The levels were ≈1% of the *act*_0567 expression amounts (7816.49 and 5082.03 RLE) under identical conditions. These results suggest that in *M. hexanoica*, *n*‐caproate is more likely liberated by CoA transfer from hexanoyl‐CoA to acetate via ACT, rather than direct hydrolysis by ACH. While further research is needed to clarify the substrate specificity of ACH, such a mechanism of ACT is consistent with the r‐BOX pathway's CoA recycling strategy. It may help maintain metabolic flux and redox balance.^[^ ^25^ ^]^ ACTs have also been studied in other chain‐elongating bacteria, such as *Clostridium tyrobutyricum* and Ruminococcaceae bacterium CPB6.^[^ ^26^ ^]^ Therefore, we performed a comparative structural analysis using AlphaFold‐predicted models. This analysis revealed that ACT_567 possesses a unique loop structure at the active site, which differs from that of the corresponding ACTs from *C. tyrobutyricum* and Ruminococcaceae CPB6. Further studies are necessary to explore these distinct conformations in more detail.

On the other hand, the tesB among the *E. coli* native acyl‐CoA thioesterases, tesB can effectively catalyze short‐chain acyl‐CoAs; ultimately, thioesterases have promiscuous activity for cleaving acyl‐CoA.^[^ ^27^ ^]^ Accordingly, its RNA expression should be precisely regulated intracellularly.^[^ ^27a^ ^]^ Likewise, ACTs in *M. hexanoica* typically maintained lower RNA expression than other r‐BOX genes, causing the concentrations of intermediates (i.e., 3‐ketohexanoyl‐CoA, crotonyl‐CoA, transenoyl‐CoA, etc.) to increase. Carboxylate production using ACTs may be more efficient than thioesterase production, as it generates one acetyl‐CoA using extracellular acetate, cleaving long‐chain acyl‐CoA to form carboxylate and CoA.^[^ ^28^ ^]^ The generated acetyl‐CoA can be used as a precursor for *n*‐caproate production.

The CoA‐transfer activity of ACTs functionally validated in engineered *E. coli* through the conversion of various C_3_–C_8_ fatty acids into their corresponding alcohols, which indicates successful formation of acyl‐CoA intermediates. When substrate specificity was observed for ACTs, the two transferases (*act*_1929 and *act*_519) favored SCCs C_3_ and C_4_. Meanwhile, the six transferases (*act*_567, *act* _347, *act*_348, *act*_2371, *act*_754, and *act*_2459) reacted with longer substrates, such as C_6_ and C_7_, and five *act*s (*act*_567, *act*_347, *act*_348, *act*_2371, and *act*_2459) converted *n‐*octanoate into *n‐*octanol (Figure 1c). Generally, ACTs undergo reversible reactions depending on the substrate concentration; a high concentration of acyl‐CoA produces corresponding carboxylates, accommodating extracellular acetate; therefore, their cooperation with ACTs with different substrate ranges incorporates extracellular C_2_–C_5_ carboxylates with CoA and releases carboxylates extended by r‐BOX. In this way, the final metabolite, *n*‐caproate, may match the maximum carbon length of the substrate released by the ACTs. This might explain why extracellular carboxylates, such as acetate and *n*‐butyrate, were continuously reduced and longer‐chain carboxylates, such as *n‐*caproate, accumulated in the fermentation experiment (Figures S5 and S6, Supporting Information). We anticipate that future studies will investigate the substrate specificity and protein structure of ACTs, contributing to the carboxylate length and providing new insights into the mechanism of chain elongation using a mixed microbiome.

*M. hexanoica* has four *acdh* genes, two of which exhibited markedly higher RNA expression than the others. Generally, the ACDH family comprises five members involved in fatty acid β‐oxidation; these are short (SCAD), medium (MCAD), long (LCAD), and very long (VLCAD1) chain acyl‐CoA dehydrogenases.^[^ ^29^ ^]^ ACDH, involved in *n*‐butyrate metabolism, is referred to as BCDH, a SCAD.^[^ ^30^ ^]^ In this study, *bcdh*_2230 from *M. hexanoica* was similar to *bcdh* from *M. elsdenii*, with typical characteristics of *bcdh* carrying *etf* αβ subunits as cofactors.^[^ ^31^ ^]^ Therefore, *bcdh*_2230 was expected to accommodate straight‐chain acyl groups containing 4–6 carbon atoms.^[^ ^30^ ^]^ However, the *E. coli* strain carrying *bcdh*_2230 did not produce *n*‐caproate regardless of cofactor co‐expression. Instead, *acdh*_2251 produced *n*‐caproate at 1.25 g L^−1^, even without introducing a cofactor gene. Compared with trans‐2‐enoyl‐CoA reductase (*ter*), *acdh*_2251 exhibited ≈1.4‐times increased *n*‐caproate production.

Trans‐2‐enoyl‐CoA reductase produces MCCs in *E. coli* without cofactors, such as *etf* αβ; however, the genetic phylogeny differed considerably from *acdh*_2251. Additionally, *acdh*_2251 maintained high RNA expression under all conditions. Hence, *acdh*_2251 is directly utilized for *n*‐caproate production and may play a role with BCDH in favoring short‐chain acyl‐CoA. However, it remains unclear how *acdh*_2251 functions in *n*‐caproate production without cofactors. When the structure of *acdh*_2251 was simulated by the AlphaFold‐predicted models, *acdh*_2251 appeared to be compatible with the native *etf* αβ of *E. coli*(PDB code: 5OL2); however, the mechanism requires further investigation through in‐depth studies.

The first attempt to synthetically produce *n*‐caproate via r‐BOX in *E. coli* identified thiolase as the key enzyme for MCC production, condensing acetyl‐CoA and butyryl‐CoA.^[^ ^14a^ ^]^ This was achieved via the *bktb* gene, which synthesizes C_4_/C_5_ polyhydroxyalkanoates (PHAs),^[^ ^32^ ^]^ resulting in *n*‐caproate production. This prompted us to hypothesize that THLs related to r‐BOX differ at the protein level. Bonk et al. presented a rational framework for increasing the selectivity ratio of thiolase, defined as the ratio of C_6_ to C_4_ PHAs. They observed that the synthesis of PHAs was highly enriched in 3HHx (C_6_) when rationally selected mutants were employed. The mutant, M158A, produced a ≈10‐fold increase in C_4_/C_6_ PHA content compared with that seen with wild‐type thiolase.^[^ ^33^ ^]^ Meanwhile, Mann and Lutke‐Eversloh (2013) generated a thiolase comprising three amino acids (R133G, H156N, and G222V) optimized for solvent production. The mutant strains delayed ethanol and *n*‐butanol formation; the ethanol and *n*‐butanol titers increased by 46% and 18%, respectively.^[^ ^34^ ^]^ These advances demonstrate the numerous research areas to explore in chain elongation.

In this study, the protein structure of *Mh*THL was determined, confirming that *n*‐caproate production is related to the size of the pocket that accepts substrates in thiolase, the first enzyme in the r‐BOX pathway. The pocket size of *Mh*THL is significantly larger than that of other THLs involved in *n‐*butyrate production. Pocket size was modulated by two amino acids, L87 and V351. Consequently, these findings provided not only a detailed enzymatic understanding of *n*‐caproate biosynthesis in *M. hexanoica* but also valuable genetic frameworks for identifying other MCC‐producing strains. The key r‐BOX genes, including *thl*_1583 and the specific residues governing substrate specificity, can serve as reliable biomarkers for genomic screening of novel MCC producers in environmental or engineered microbiomes. We propose that these critical residues could be used as genetic indicators in MCC production using open cultures and as selection markers for *n*‐caproate‐producing bacteria. Additionally, mutants of these residues could be used to increase carboxylate size or *n*‐caproate production. Although key residues constituting the substrate‐binding pocket in *Mh*THL have been identified, determination of acyl‐CoA‐bound structures at atomic resolution may be necessary for precise specificity engineering of thiolases. Enzymatic hydrolysis side reactions hinder such structural determination.^[^ ^35^ ^]^

Although our soaked structure was obtained using the C88S variant, it is unclear why hydrolyzed CoA is bound, and it is still possible that the cysteine‐to‐serine alteration did not completely eliminate hydrolysis reactivity. We anticipate that simultaneous application of co‐crystallization/soaking methods using non‐hydrolysable substrates and enzymes with relatively low hydrolytic activity could enable the determination of such structures, thereby contributing to thiolase engineering for higher carbon condensation.

The technological implications of this study can extend to feasible applications to sustainable bio‐derived chemicals and fuels production. MCCAs' lengths could be controlled selectively by the lengths of short‐chain carboxylates (C_3_‐C_5_) in *M. hexanoica*. Notably, the addition of acetate facilitated the metabolic pathway that transfers CoA from hexanoyl‐CoA, thereby promoting both *n*‐caproate production and microbial growth. These features provide strategies to boost MCCs production even in open‐source culture or in pure culture using other chain elongating species. Furthermore, when core genes related to *n*‐caproate were observed through RNA expression, the ACTs participated in the liberation of *n*‐caproate, and their expressions were regulated relatively lower (e.g., *act*s: 5082.03 RLE). Notably, ACTs were proposed as more effective enzymes than acetyl‐CoA hydrolase because they liberate *n*‐caproate by converting acetate into acetyl‐CoA. These genes can be used effectively not only for MCCs but also for the production of valuable chemicals. Ultimately, the study highlighted the structural characteristics of *Mh*THL, which is the most important enzyme for the synthesis of *n*‐caproate. *Mh*THL had a relatively bigger substrate‐accommodating pocket involved in *n*‐caproate production, and two amino acids determined the size. These findings enable the easy identification and distinction of MCC‐producing microbes using common molecular tools like PCR or qPCR. These technical applications will be beneficial for advancing bio‐industries toward sustainable chemicals and energies.

Currently, prediction tools for protein structures have been developed using deep learning algorithms, with empirical data simultaneously accumulated.^[^ ^36^ ^]^ These technical advancements can be utilized in our structural improvement of thiolase and can be applied to enhance selectivity and productivity with empirical data simultaneously accumulated.^[^ ^36b–d^ ^]^ Furthermore, incorporating metabolomics analyses could complement our genetic and transcriptomic findings, offering a more comprehensive validation of the caproate synthesis pathway. This integrative approach would not only increase the rigor and credibility of our research but also provide deeper insights into the chain elongation mechanism. These future research directions can broaden our knowledge of the chain elongation mechanism and bio‐derived chemical production, moving us one step closer to a sustainable society without the use of fossil‐derived chemicals.

## 4 Experimental Section

### Strain, Culture Conditions, and *n*‐Caproate Production using Extractive Fermentation

*Megasphaera hexanoica* was isolated by Jeon et al.^[^ ^37^ ^]^ and cultivated in modified peptone yeast extract‐fructose medium (mPYF from DSM 104 medium).^[^ ^38^ ^]^ To investigate the electron‐acceptor effect of *M. hexanoica* toward short‐ to medium‐chain carboxylates, batch cultures were supplemented with various C_3_–C_6_ carboxylates (0.1 m final concentration), including propionate, *n*‐butyrate, and *n*‐valerate, along with 0.1 m acetate. For *n*‐caproate (C6), 0.05 m was added to minimize toxicity. In all cases, 0.1 m fructose was provided as the electron donor.

A set of control conditions was also tested: 1) fructose only, without external carboxylates; 2) fructose with 0.1 m acetate only; 3) 0.1 m acetate without fructose; and 4) the modified peptone yeast extract (mPY, from DSM 104 medium) without any added carboxylates and fructose. Details on substrate consumption and product formation were provided in Table S1 (Supporting Information), and the experimental design was illustrated in Figure S1 (Supporting Information). To achieve optimized production for *n*‐caproate, three culture condition parameters (i.e., acetate concentration, *n*‐butyrate concentration, and pH) were adjusted using the response surface methodology to optimize *n*‐caproate production. The experimental plan to determine the optimum conditions was designed according to the central composite design (CCD) (Table S2, Supporting Information). The optimum conditions were 8 g L^−1^ sodium acetate, 14 g L^−1^ sodium butyrate, and pH 6.2. In general, the volume of culture used was 20, and 50‐mL serum bottles were used. Extractive fermentation under the same culture conditions was conducted in a fermenter with a 3‐L working volume, integrated with the extraction process using a mixed solvent with a 9:1 ratio of oleyl alcohol (Ecogreen Oleochemicals GmbH, Dessau‐Roßlau, Germany) and Alamine 336 (Cognis GmbH, Monheim, Germany) (Figure S5, Supporting Information). A detailed description of extractive fermentation has been provided elsewhere.^[^ ^39^ ^]^

Plasmid construction was performed in *E. coli* grown in Luria‐Bertani (LB) broth (10 g L^−1^ tryptone, 5 g L^−1^ yeast extract, and 10 g L^−1^ NaCl). The platform strain performance was evaluated in LB medium supplemented with 20 g L^−1^ glucose. Antibiotics were added to the markers on the respective plasmids (pGS21a: 50 mg L^−1^ ampicillin, pCOLA duet: 50 mg L^−1^ kanamycin, and pCDF duet: 100 mg L^−1^ spectinomycin). The plasmid pGS21a was obtained from GenScript (Piscataway, NJ, USA). The plasmid pCOLA duet and pCDF duet were purchased from Merck‐Novagen (Novagen, Madison, WI, USA). Kanamycin was purchased from GoldBio (GoldBio, St Louis, MO, USA). Ampicillin and spectinomycin were purchased from Sigma–Aldrich (Sigma–Aldrich Korea, Ltd.). Evaluation of *n*‐caproate production by the engineered *E. coli* strains was performed in Terrific broth supplemented with 20 g L^−1^ glucose.^[^ ^40^ ^]^ The initial pH was adjusted to 7.0 using 3m NaOH. Isopropyl β‐D‐1‐thiogalactopyranoside (IPTG; 1 mm) was added as an inducer during the log phase (OD_600nm_ = 0.8). All compounds not listed here were purchased from Sigma–Aldrich (Sigma–Aldrich Korea, Ltd.)

### Whole Genome Analysis

*M. hexanoica* was cultivated in 20 mL of anoxic PYF medium containing 20 g L^−1^ of fructose at 37 °C and 150 rpm. Genomic DNA was extracted using the G‐spin genomic DNA extraction kit from iNtRON Biotechnology Inc., Seoul, Korea, according to the provided instructions. The genome was sequenced using both an Illumina Hiseq system with a 150‐base pair (bp) paired‐end library and a 454 Genome Sequencer FLX Titanium system (Roche, Basel, Switzerland) with an 8‐kb paired‐end library. The Illumina reads were assembled using CLC Genomics Workbench ver. 5.0 (QIAGEN, Valencia, CA, USA). The initial assembly was adapted for the CLC Genomics Workbench by creating fake reads from the consensus to gather the read pairs in the Illumina paired‐end library. The 454 paired‐end reads were assembled with Illumina data using gsAssembler ver. 2.6 (Roche). Gaps between the contigs were partially filled by sequencing Polymerase Chain Reaction (PCR) products, resulting in one scaffold with nine contigs. CodonCode Aligner (CodonCode Corp., Dedham, MA, USA) and CLC Genomics Workbench 5.0 were employed for sequence assembly and quality assessment in the subsequent finishing process. The Illumina (220.08‐fold coverage; 4194510 reads) and 454 sequencing (33.06‐fold coverage; 442469 reads) platforms provided 12× coverage of the genome. The final assembly identified one contig. Annotation was conducted using NCBI Prokaryotic Genome Annotation Pipeline v3.0 (https://www.ncbi.nlm.nih.gov/refseq/annotation_prok/). Whole genome data were deposited in the NCBI database under GenBank number (CP011940.1). The assembled genome was annotated using the EzBioCloud annotation pipeline.^[^ ^41^ ^]^

### RNA Transcriptomic Analysis

To isolate total RNA, *M. hexanoica* was cultured under the following conditions. *Megasphaera hexanoica* was cultivated in a mPYF medium, and the optimum conditions acquired from RSM were used as mentioned earlier. For transcriptomic analysis, cultures were grown under two different conditions: 1) a production condition (AB) with 8 g L^−1^ sodium acetate and 14 g L^−1^ butyrate supplementation, and 2) a non‐production condition (N) without supplementation. Samples were collected at 9 h (exponential phase) and 18 h (stationary phase), and labeled as AB9, AB18, N9, and N18. To rapidly quench cellular metabolism and preserve RNA integrity, an equal volume of methanol prechilled to −70 °C was added immediately to each culture sample. RNA extractions were carried out simultaneously for all conditions to ensure consistency in processing. Total RNA of *M. hexanoica* was extracted manually to identify the target genes.^[^ ^42^ ^]^ RNA samples were subjected to four combinatorial conditions for growth time and *n*‐caproate production. The conditions were divided into two groups: *n*‐caproate production and non‐production conditions. Two time points (9 and 18 h after inoculation) were sampled for each condition. The first time point represented the initial growth. After 18 h of incubation, the cultures were considered stationary. The conditions for *n*‐caproate production were determined by adding SCCs. Samples were sequenced with the Illumina Genome Analyzer Iix (Illumina, Inc., San Diego, CA, USA) to generate nondirectional, single‐ended 36‐base pair reads. Quality‐filtered reads were annotated to the reference genome sequence using CLC Genomics Workbench (version 5.0; CLCbio, Katrinebjerg, Denmark). Four runs of RNA‐seq resulted in 21.8m spots, 783.3M bases, and 225.5‐Mb size. The run's processing numbers were SRR2075767, SRR2075768, SRR2075769, and SRR2075770. The relative transcription was calculated by numbering the reads per kilobase of the exon model per million mapped sequence reads (RPKM) and the relative log expression. Transcriptional data were deposited in the NCBI database under the accession numbers (SRP059812 and PRJNA287855).

### Selection of Effective Genes Related to *n*‐Caproate Production

Genes related to *n*‐caproate synthesis were selected by analyzing the *M. hexanoica* genome using CL Genomic software (ChunLab Inc., Seoul, Korea) and the KEGG mapper.^[^ ^43^ ^]^ Twenty genes associated with carboxylate and coenzyme A production were identified. The respective genes were aligned according to their expected chemical reactions, and the postulated metabolic pathways are shown in Figure 1.

Effective genes were selected using the transcriptomic results. Expression levels were evaluated using CLRNAseq software (ChunLab Inc.). Genes related to coenzyme A production or the r‐BOX pathway exhibited higher expression levels (Table S8, Supporting Information). The genes were *thl* (*Mh*_1583), *hbd* (*Mh*_2207), *crt (Mh*_2206), *acdh* (*Mh*_2251), *bcdh*‐*etfαβ* (*Mh*_2228‐2230), and *act* (*Mh*_567) (Table S9, Supporting Information). The platform was developed using the pCDF duet plasmid (Novagen). The *adhe*2 gene from *C. acetobutylicum* ATCC 824 was inserted into the multiple cloning site (MCS) 2 of the pCDF duet plasmid using NdeI and XhoI restriction sites. The *adhe*2 contains a potential region for digestion by NdeI; this region was mutated to prevent digestion. The *act* genes were inserted into the MCS‐2 site of the pCDF duet plasmid (Table S10, Supporting Information). To express *act* genes at the same level, the 5′‐untranslted region of ACTs was designed using the UTR designer in silico (https://sbi.postech.ac.kr/utr_designer/) (Tables S6 and S7, Supporting Information).

As a host strain, *E. coli* MG1655 DE3 (△*ldh*, △*adhE*, △*frdA*, and △*pta*) donated by Oh et al. was utilized for expression.^[^ ^44^ ^]^ Terrific broth medium containing 20 g L^−1^ glucose was used to evaluate ACT enzyme function. The C_3_–C_6_ carboxylates (1 g L^−1^) were added as precursors for C_3_–C_6_ alcohol production; 1 mm was added to express the target protein at an OD_600_ of 0.8.

### Platform Strain and Plasmid Construction

The strains and plasmids used for the platform are listed in Tables S10 and S12 (Supporting Information). Genes from *M. hexanoica* and the NCBI GenBank were inserted into the constructed plasmids to evaluate the functions of *thl*. Seven *M. hexanoica* genes with high RNA expression levels were identified: *hbd, crt*, *acdh*, *bcdh*, *etf* αβ, and *act*. In addition, trans‐enoyl‐CoA reductase from *T. denticola* was evaluated. *hbd* and *crt* from *M. hexanoica* were inserted into MCS‐1 of the pCDFDuet‐1 plasmid using NcoI and NotI restriction sites. The *bcdh*‐*etf* αβ gene cluster was inserted into the MCS‐2 of pCDFDuet‐1. *acdh* was inserted upstream of *bcdh* using the NdeI restriction site. Additionally, a transenoyl‐CoA reductase (TER, FabV, NCBI Gene ID 2 741 560, and EC:1.3.1.44) from *T. denticola* was inserted into the cluster comprising *bcdh*, *etf* αβ, *acdh*, and *act*. The *ter* was codon‐optimized and synthesized using GenScript (GenScript Inc.). The *thl* gene from *M. hexanoica* was independently inserted into the pCOLADuet‐1 plasmid using the NdeI and XhoI restriction sites. To compare the function of thiolases from various strains, codons of different *thl* genes were optimized and synthesized using GenScript (GenScript Inc.).

### Comparison of Thiolases

The impact of five thiolases (*Mh*thl, *Ck*thl, *Cac*thl, *Ec*atoB, and *Re*bktB) from *M. hexanoica*, *C. kluyveri*, *C. acetobutylicum*, *E. coli*, and *R. eutropha* on *n*‐caproate production was analyzed. All *thl* genes were codon‐optimized, synthesized by GenScript (GenScript Inc.), and inserted at identical restriction sites (NdeI and XhoI) of the pCOLADuet‐1 plasmid. Expression of these genes was evaluated in *E. coli* BL21 DE3 cells. Overexpression was achieved by IPTG supplementation. Plasmids encoding the respective *thl* were inserted into *E. coli* MG1655 DE3 (△*ldh*, △*adhE*, △*frdA*, and △*pta*) carrying the plasmid with *hbd, crt, acdh*, and *act* (Figure S13, Supporting Information). Genes were expressed in *E. coli; n*‐caproate production was compared simultaneously.

### Analytical Methods

The carboxylates and alcohols were analyzed using a gas chromatograph equipped with a Stabiliwax–DA column (Restek Corp., Bellefonte, PA, USA) and a flame ionization detector (Agilent 7890; Agilent Technologies Inc., Santa Clara, CA, USA). The analysis conditions were set to 3 min/hold at 80 °C and 1 min/(30–190 °C), and 3 min/hold at 190 °C.^[^ ^45^ ^]^ Tracking of carboxylic acid isotopes was conducted using a gas chromatograph/time‐of‐flight mass spectroscopy (GC/TOF‐MS, Pegasus III, Leco Co., St. Joseph, MI, USA) instrument equipped with a 6890N GC (Agilent Technologies Inc.) and an HP‐INNOWax column (30‐m length × 0.25‐mm I.D. × 0.25‐µm thickness). The oven temperature was programmed to increase from 50 to 190 °C at 10 °C min^−1^, held for 2 min at 190 °C, and increased to a final temperature of 240 °C at a rate of 15 °C min^−1^. The TOF‐MS condition was as follows: The mass scan rate was 10 spectra/min from 40 to 200 m z^−1^. The ion source was 230 °C. Carbon consumption was analyzed using a high‐performance liquid chromatograph connected to a refractive index detector (Agilent 1100 series; Agilent Technologies Inc.). The analyzed samples were run on a Hi‐plex H column (Agilent Technologies Inc.).^[^ ^10^ ^]^ Five millimolar sulfuric acid was used as the mobile phase, and the flow rate was set to 0.6 mL min^−1^.

### *Mh*THL Preparation

Plasmid construction, expression, purification, and crystallization of *Mh*THL were performed as described by Kim et al. (2015).^[^ ^46^ ^]^ The *Mh*THL coding gene (Met1–Asp396, 40.5 kDa) was amplified by PCR using *Mh*THL chromosomal DNA as the template with *pfu* polymerase (Solg Pfu‐X DNA Polymerase; SolGent Co., Ltd., Daejeon, Korea). The PCR product was subcloned into pProEX HTa (Life Technologies, Gaithersburg, MD, USA) with 6×‐Histag and rTEV at the N‐terminus. The expression construct was transformed using an electroporator (Gene Pulser Xcell Electroporation Systems; Bio‐Rad Laboratories Inc., Hercules, CA, USA) into an *E. coli* BL21 (DE3) strain grown in 1 L of LB medium containing ampicillin (50 mg mL^−1^) at 150 rpm and 37 °C. Cells were induced by adding IPTG at a final concentration of 1 mM. Cultures were further maintained at 18 °C for 20 h and harvested by centrifugation at 5000 × *g* and 4 °C. The resulting cell pellet was resuspended in buffer A (40 mM Tris‐HCl at pH 8.0 and 5 mM β‐mercaptoethanol) and disrupted by ultrasonication. Cell debris was removed by centrifugation at 11000 × *g* and 4 °C for 1 h, and the proteins in the lysate were bound to Ni‐NTA agarose (QIAGEN Korea, Co., Ltd, Seoul, Korea). After washing with buffer A containing 20 mM imidazole, the bound proteins were eluted with 300 mM imidazole in buffer A. HiLoad 26/60 Superdex 200 Prep Grade (GE Healthcare Bio‐Sciences, Pittsburgh, PA, USA) size exclusion chromatography was performed to remove trace amounts of contamination. The protein purity was > 95%, as determined by SDS‐PAGE, and concentrated to 42 mg mL^−1^ in 40 mM Tris‐HCl (pH 8.0) and 1 mM dithiothreitol. To produce *Mh*THL mutant proteins, PCR‐based site‐directed mutagenesis experiments were performed using a primer containing one mismatched sequence, and the mutant proteins were purified using a procedure similar to that used for wild‐type *Mh*THL.

### Crystallization, Data Collection, and Structure Determination of *Mh*THL

The crystals for the diffraction experiments were obtained using the hanging‐drop vapor diffusion method. The drop contained 1.0 µL of the purified enzyme mixed with 1.0 µL of reservoir solution. The crystals of *Mh*THL appeared under a reservoir condition of 25% w/v polyethylene glycol (PEG)‐3350 and 0.1 M HEPES at pH 7.5 for 7 days at 22 °C. The crystals were transferred to a cryoprotectant solution containing 25% w/v PEG‐3350, 0.1 M HEPES pH 7.5, and 20% (v/v) glycerol. They were soaked in the solution supplemented with 25 mM hexanoyl‐CoA for 5 min at room temperature and flash‐frozen by immersion at −173 °C in liquid nitrogen to provide a more detailed description of the related procedure. The data were collected to a resolution of 1.64 Å at 7 A beamline of the Pohang Accelerator Laboratory (Pohang, Korea) using an ADSC Quantum 270 CCD detector (Area Detector Systems, Poway, CA, USA). The data were processed using XDS^[^ ^47^ ^]^ and aimless of CCP4^[^ ^48^ ^]^ and merged to a space group *P*22_1_2_1_, with unit cell parameters of *a =* 51.51 Å, *b =* 111.44 Å, *c =* 140.99 Å, and α = β = γ = 90°. The structure of *Mh*THL was determined by molecular replacement with the CCP4 version of MOLREP^[^ ^49^ ^]^ using the *Re*BktB structure (Protein Data Bank (PDB) code 4NZS) as a search model. Model building was performed manually using WinCoot,^[^ ^50^ ^]^ and refinement was performed using CCP4 refmac5^[^ ^51^ ^]^ and CNS.^[^ ^52^ ^]^ The refined model was deposited in the PDB code 8JG2. All statistical analyses are summarized in Table S13 (Supporting Information).

### Statistical Analysis

To ensure statistical consistency across all experiments, the type of electron acceptor (short‐chain carboxylic acids), bottle size (60 mL), and working volume (20 mL) were standardized. All experiments were performed in duplicate (*n* = 2), and results were presented as mean values ± standard deviation (SD). Data for Table S1 (Supporting Information) (electron acceptors ranging from C2 to C8) were summarized as mean ± SD using Microsoft Excel (Microsoft, Redmond, WA, USA). Experiments using electron acceptors ranging from C_2_ to C_8_ were conducted under these unified conditions, and the corresponding data were summarized in Table S1 (Supporting Information).

To evaluate multifactorial effects on *n*‐Caproate production, a response surface methodology (RSM) based on a central composite design (CCD) was employed. Analysis of variance (ANOVA) was used to assess the significance of model terms and interactions, and model adequacy was confirmed by the coefficient of determination (R^2^ and adjusted R^2^). Post‐hoc comparisons between different factor levels were conducted using Tukey's Honest Significant Difference (HSD) test. Statistical analyses for RSM, ANOVA, and post‐hoc comparisons were performed using Design‐Expert software (Stat‐Ease Inc., Minneapolis, MN, USA), and visualizations of post‐hoc comparisons were conducted using Python (pandas, statsmodels, seaborn, and matplotlib).

To assess statistical differences, all other paired comparisons were conducted using Origin 2025 software (OriginLab, Northampton, MA, USA). In the case of *n*‐caproate production by genetically engineered *E. coli*, eight different gene combinations were constructed, and paired *t*‐test were conducted to evaluate statistical significance between each combination (Figure 2). For thiolase activity, paired comparisons were conducted between each of the five thiolases derived from different microbial strains or the two *Mh*THL‐derived mutants and a reference thiolase (Figure 3). Statistical significance was determined at a threshold of *p* < 0.05. Significant differences between experimental groups were indicated using asterisks (^*^), where *p* < 0.05 was denoted by ^*^, *p* < 0.01 by ^**^, and *p* < 0.001 by ^***^.

To minimize environmental variability and ensure consistent experimental conditions in metabolic engineering experiments using *E. coli*, several measures were taken. The same plasmid backbone was used across all constructs, with insertion at consistent restriction enzyme sites whenever possible. Induction conditions were standardized by applying identical 1 mm IPTG concentrations and sampling times. Additionally, all heterologous genes were codon‐optimized for *E. coli* to promote uniform expression levels. These strategies were implemented to enhance reproducibility and allow for meaningful comparisons among different gene combinations.

## Conflict of Interest

The authors declare no conflict of interest.

## Author Contributions

B.S.J. and E.J.K. contributed equally to this work. B.S.J. and E.J.K. conducted the experiments and wrote the manuscript draft. H.K. contributed to the study design and helped write the manuscript and construct the figures. Seo solved the protein structure data using computational methods and wrote parts of the thiolase structure. S.J.S. cooperated with B.S.J. to construct the *E. coli* platform. L.T.A. discussed the results with the authors and advised on writing the manuscript, in addition to extensive editing. Caroline Schlaiß discussed the manuscript with B.S.J. and assisted in its writing. K.J.K. and B.I.S. supervised the experimental design and provided advice on writing the manuscript.

## Supporting information



Supporting Information

## Data Availability

The data that support the findings of this study are available from the corresponding author upon reasonable request.

## References

[1] a) V. Elhami , E. C. Antunes , H. Temmink , B. Schuur , Molecules 2022, 27, 1389; 35209179 10.3390/molecules27041389 PMC8877087

[2] H. Wang , Y. Gu , W. Zhou , D. Zhao , Z. Qiao , J. Zheng , J. Gao , X. Chen , C. Ren , Y. Xu , Appl. Environ Microbiol. 2021, 87, 0120321. 10.1128/AEM.01203-21 PMC8478455 34378978

[3] H. C. Bailey , R. G. W. Norrish , Series A. Mathematical and Physical Sciences 1952, 212, 311.

[4] Q. Wu , X. Bao , W. Guo , B. Wang , Y. Li , H. Luo , H. Wang , N. Ren , Biotechnol. Adv. 2019, 37, 599. 30849433 10.1016/j.biotechadv.2019.03.003

[5] V. De Groof , M. Coma , T. Arnot , D. J. Leak , A. B. Lanham , Molecules 2019, 24, 398. 30678297 10.3390/molecules24030398 PMC6384945

[6] C. Urban , J. Xu , H. Sträuber , T. R. dos Santos Dantas , J. Mühlenberg , C. Härtig , L. T. Angenent , F. Harnisch , Energy Environ. Sci. 2017, 10, 2231.

[7] J. Wang , Y. Yin , Biotechnol. Adv. 2022, 55, 107882. 34871718 10.1016/j.biotechadv.2021.107882

[8] a) L. Jiang , H. Fu , H. K. Yang , W. Xu , J. Wang , S. T. Yang , Biotechnol. Adv. 2018, 36, 2101; 30266343 10.1016/j.biotechadv.2018.09.005

[9] a) B. S. Jeon , S. Kim , B. I. Sang , Int. J. Syst. Evol. Microbiol. 2017, 67, 2114; 28742009 10.1099/ijsem.0.001888

[10] B. S. Jeon , S. Kim , B. I. Sang , Int. J. Syst. Evol. Micr. 2017, 67, 2114. 10.1099/ijsem.0.001888 28742009

[11] a) K. J. Steinbusch , H. V. Hamelers , C. M. Plugge , C. J. J. E. Buisman , Energy Environ. Sci. 2011, 4, 216;

[12] a) S. G. Kim , S. Jang , J. H. Lim , B. S. Jeon , J. Kim , K. H. Kim , B. I. Sang , G. Y. Jung , Bioresour. Technol. 2018, 247, 1253; 29054557 10.1016/j.biortech.2017.10.014

[13] N.‐R. Lee , C. H. Lee , D.‐Y. Lee , J.‐B. Park , Microorganisms 2020, 8, 539.32283671

[14] a) Y. Dekishima , E. I. Lan , C. R. Shen , K. M. Cho , J. C. Liao , J. Am. Chem. Soc. 2011, 133, 11399; 21707101 10.1021/ja203814d

[15] a) G. N. Bennett , F. B. Rudolph , FEMS Microbiol. Rev. 1995, 17, 241;

[16] H. Barker , M. Kamen , B. T. Bornstein , Proc. Natl. Acad. Sci. USA 1945, 31, 373. 16588706 10.1073/pnas.31.12.373 PMC1078850

[17] a) Y. Cheon , J. S. Kim , J. B. Park , P. Heo , J. H. Lim , G. Y. Jung , J. H. Seo , J. H. Park , H. M. Koo , K. M. Cho , J. B. Park , S. J. Ha , D. H. Kweon , J. Biotechnol. 2014, 30, 182; 10.1016/j.jbiotec.2014.04.010 24768798

[18] a) S. Atsumi , A. F. Cann , M. R. Connor , C. R. Shen , K. M. Smith , M. P. Brynildsen , K. J. Chou , T. Hanai , J. C. Liao , Metab. Eng. 2008, 10, 305; 17942358 10.1016/j.ymben.2007.08.003

[19] S. Kim , Y. S. Jang , S. C. Ha , J. W. Ahn , E. J. Kim , J. H. Lim , C. Cho , Y. S. Ryu , S. K. Lee , S. Y. Lee , K. J. Kim , Nat. Commun. 2015, 6, 8410. 26391388 10.1038/ncomms9410 PMC4595758

[20] Y. Modis , R. K. Wierenga , J. Mol. Biol. 2000, 297, 1171. 10764581 10.1006/jmbi.2000.3638

[21] T. R. Kiema , R. K. Harijan , M. Strozyk , T. Fukao , S. E. Alexson , R. K. Wierenga , Acta. Crystallogr. D Biol. Crystallogr. 2014, 70, 3212. 25478839 10.1107/S1399004714023827

[22] A. C. Marshall , C. S. Bond , J. B. Bruning , ACS Catal. 2018, 8, 1973.

[23] Y. Tao , X. Zhu , H. Wang , Y. Wang , X. Li , H. Jin , J. Rui , J. Biotechnol. 2017, 259, 91. 28774671 10.1016/j.jbiotec.2017.07.036

[24] a) F. Garces Daza , F. Haitz , A. Born , E. Boles , Biotechnol. Biofuels Bioprod. 2023, 16, 71; 37101299 10.1186/s13068-023-02317-z PMC10134560

[25] a) H. Kim , S. Kang , B. I. Sang , Bioresour. Technol. 2022, 344, 126211. 34710599 10.1016/j.biortech.2021.126211

[26] Q. Yang , S. Guo , Q. Lu , Y. Tao , D. Zheng , Q. Zhou , J. Liu , Biosci. Rep. 2021, 41, BSR20211135. 34338280 10.1042/BSR20211135 PMC8360832

[27] a) J. H. Lim , S. W. Seo , S. Y. Kim , G. Y. Jung , Bioresour. Technol. 2013, 135, 568; 23127832 10.1016/j.biortech.2012.09.091

[28] M. Sato , Y. Yoshida , K. Nagano , Y. Hasegawa , J. Takebe , F. Yoshimura , Front. Microbiol. 2016, 7, 1146. 27486457 10.3389/fmicb.2016.01146 PMC4949257

[29] J. J. Kim , R. Miura , Eur. J. Biochem. 2004, 271, 483. 14728675 10.1046/j.1432-1033.2003.03948.x

[30] G. Williamson , P. C. Engel , Biochem. J. 1984, 218, 521. 6712628 10.1042/bj2180521 PMC1153368

[31] S. Djordjevic , C. P. Pace , M. T. Stankovich , J. J. Kim , Biochemistry 1995, 34, 2163. 7857927 10.1021/bi00007a009

[32] H. B. Machado , Y. Dekishima , H. Luo , E. I. Lan , J. C. Liao , Metab. Eng. 2012, 14, 504. 22819734 10.1016/j.ymben.2012.07.002

[33] B. M. Bonk , Y. Tarasova , M. A. Hicks , B. Tidor , K. L. J. Prather , Biotechnol. Bioeng. 2018, 115, 2167. 29877597 10.1002/bit.26737 PMC6131064

[34] M. S. Mann , T. Lutke‐Eversloh , Biotechnol. Bioeng. 2013, 110, 887. 23096577 10.1002/bit.24758

[35] a) S. Masamune , M. A. Palmer , R. Gamboni , S. Thompson , J. T. Davis , S. F. Williams , O. P. Peoples , A. J. Sinskey , C. T. Walsh , J. Am. Chem. Soc. 1989, 111, 1879;

[36] a) S. K. Burley , H. M. Berman , Structure 2021, 29, 515; 33984281 10.1016/j.str.2021.04.010 PMC8178243

[37] B. S. Jeon , O. Choi , Y. Um , B. I. Sang , Biotechnol. Biofuels 2016, 9, 129. 27340431 10.1186/s13068-016-0549-3 PMC4918077

[38] DSMZ. PYG MEDIUM (modified), Media ‐ MediaDive. https://mediadive.dsmz.de/medium/104 (accessed 2025‐09‐06).

[39] K. Choi , B. S. Jeon , B. C. Kim , M. K. Oh , Y. Um , B. I. Sang , Appl. Biochem. Biotechnol. 2013, 171, 1094. 23754557 10.1007/s12010-013-0310-3

[40] K. D. Tartoff , C. A. Hobbs , Bethesda Res. Lab. 1987, 9, 12.

[41] S.‐H. Yoon , S.‐M. Ha , S. Kwon , J. Lim , Y. Kim , H. Seo , J. Chun , Int. J. Syst. Evol. Microbiol. 2017, 67, 1613. 28005526 10.1099/ijsem.0.001755 PMC5563544

[42] M. Ares , Cold Spring Harbor Protoc. 2012, 9, 1024. 10.1101/pdb.prot071068 22949721

[43] M. Kanehisa , S. Goto , Y. Sato , M. Furumichi , M. Tanabe , Nucleic Acids Res. 2011, 40, D109. 22080510 10.1093/nar/gkr988 PMC3245020

[44] J. M. Baek , S. Mazumdar , S. W. Lee , M. Y. Jung , J. H. Lim , S. W. Seo , G. Y. Jung , M. K. Oh , Biotechnol. Bioeng. 2013, 110, 2790. 23568786 10.1002/bit.24925

[45] B. S. Jeon , C. Moon , B. C. Kim , H. Kim , Y. Um , B. I. Sang , Enzyme Microb. Technol. 2013, 53, 143. 23830453 10.1016/j.enzmictec.2013.02.008

[46] S. Kim , Y.‐S. Jang , S.‐C. Ha , J.‐W. Ahn , E.‐J. Kim , J. Hong Lim , C. Cho , Y. Shin Ryu , S. Kuk Lee , S. Y. Lee , K. J. Kim , Nat. Commun. 2015, 6, 8410. 26391388 10.1038/ncomms9410 PMC4595758

[47] W. Kabsch , Acta. Crystallogr. D Biol. Crystallogr. 2010, 66, 133. 20124693 10.1107/S0907444909047374 PMC2815666

[48] M. D. Winn , C. C. Ballard , K. D. Cowtan , E. J. Dodson , P. Emsley , P. R. Evans , R. M. Keegan , E. B. Krissinel , A. G. Leslie , A. McCoy , S. J. McNicholas , G. N. Murshudov , N. S. Pannu , E. A. Potterton , H. R. Powell , R. J. Read , A. Vagin , K. S. Wilson , Acta. Crystallogr. D Biol. Crystallogr. 2011, 67, 235. 21460441 10.1107/S0907444910045749 PMC3069738

[49] A. Vagin , A. Teplyakov , Acta. Crystallogr. D 2010, 66, 22. 20057045 10.1107/S0907444909042589

[50] P. Emsley , K. Cowtan , Acta. Crystallogr. D 2004, 60, 2126. 15572765 10.1107/S0907444904019158

[51] G. N. Murshudov , A. A. Vagin , E. J. Dodson , Acta. Crystallogr. D 1997, 53, 240. 15299926 10.1107/S0907444996012255

[52] A. T. Brunger , P. D. Adams , G. M. Clore , W. L. DeLano , P. Gros , R. W. Grosse‐Kunstleve , J. S. Jiang , J. Kuszewski , M. Nilges , N. S. Pannu , R. J. Read , L. M. Rice , T. Simonson , G. L. Warren , Acta. Crystallogr. D 1998, 54, 905. 9757107 10.1107/s0907444998003254

