# Supplementary material for: Molecular Chain Elongation Mechanism for n‐Caproate Biosynthesis by Megasphaera Hexanoica
Source: Adv Sci (Weinh). 2025 Sep 11;12(44):e06069. doi: 10.1002/advs.202506069 (PMC12667485; doi:10.1002/advs.202506069)
Supplement: Supplementary file 1 — Supporting Information [file ADVS-12-e06069-s001.docx]

Supporting Information

Molecular Chain Elongation Mechanism for *n*-Caproate Biosynthesis by *Megasphaera hexanoica*

Byoung Seung Jeon^†^, Eun-Jung Kim^†^, Hogyun Seo, Hyunjin Kim, Seungjin Shin, Caroline Schlaiß, Largus T. Angenent, Kyung-Jin Kim*, and Byoung-In Sang*

**Supplementary Text**

**Effect of Short-Chain Carboxylate (SCC) Supplementation on *Megasphaera hexanoica* Carbon Chain Elongation**

Here, the effect of SCC supplementation was evaluated to determine whether *M. hexanoica* creates longer-chain carboxylates by elongating them by two carbons from the electron donor fructose. Furthermore, the medium-chain carboxylate *n*-caproate was added to determine whether it is elongated into *n*-caprylate (C_8_ carboxylate). Acetate was added in all conditions except condition A (**Figure S1a**). The added carboxylate concentration was set to 0.1 M; however, *n*-caproate (0.05 M) was added under condition B (**Figure S1e** and **Table S1**). Fructose (0.1 M) was added as the electron donor under all conditions. Without extracellular carboxylates, *M*. *hexanoica* produced 0.9 g/L *n*-caproate from fructose (**Figure S1a**). After adding 0.1 M (7.4 g/L) propionate, *M. hexanoica* produced 5.74 g/L, correlating with an acetate consumption of ~4.37 g/L (**Table S1** and **Figure S1b**). When *n*-valerate (C_5_ carboxylate) was added as the electron acceptor, the cells produced 6.33 g/L *n*-valerate and 3.56 g/L *n*-heptanoate. *n*-Caprylate (1.15 g/L) was produced when the cultures were supplemented with 0.1 M acetate and 0.05 M *n*-caproate. Thus, *M. hexanoica* followed the chain elongation mechanism rules, in which carboxylates are elongated by two carbons at a time from the carboxyl atom at position 1 (carboxylic group). Detailed information on the carboxylates consumed and produced is presented in **Table S1**.


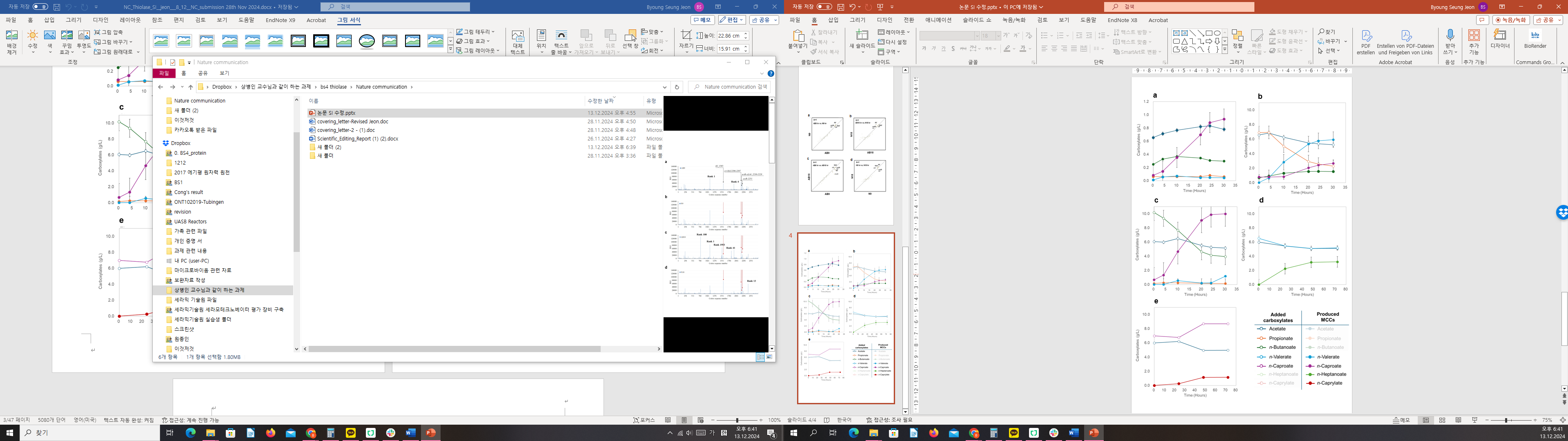


**Figure S1.** Longer-chain carboxylates are produced by *M. hexanoica* by adding fructose or fructose with various carboxylates: **a** without external addition of electron acceptors; **b** with propionate and acetate supplementation; **c** with principal acetate and *n-*butyrate supplementation; **d** with acetate and *n-*valerate supplementation; **e** with acetate and *n-*caproate supplementation. Fructose was added as an electron donor in all conditions; consumed amounts are displayed in **Table S1**. Data in **Figure S1** represent the mean ± standard deviation (SD) of two technical replicates for each sample, calculated using Origin software.

**Table S1**. Metabolites and cell growth in the presence of various extracellular carboxylates in *Megasphaera* *hexanoica*

| **Culture**  **media** | **Metabolite and electron acceptors in aqueous phase (g/L)** | | | | | | | | | | | | | | **C_2_-C_8_ ^d^** | | **Consumed fructose** | | **Metabolite in gas phase (mM)** | | | | **OD _600nm_** | | **pH** | |  |
| --- | --- | --- | --- | --- | --- | --- | --- | --- | --- | --- | --- | --- | --- | --- | --- | --- | --- | --- | --- | --- | --- | --- | --- | --- | --- | --- | --- |
|  | **Acetate** | | ***n-*Propionate** | | ***n-*Butyrate** | | ***n-*Valerate** | | ***n-*Caproate** | | ***n-*Heptanoate** | | ***n-*Caprylate** | | **(g/L)** | | **(g/L)** | | **H_2_** | | **CO_2_** | |  |  |  |  |  |
| **RCM ^c^** | | 0.33 | | 0.08 | | 0.39 | | 0.1 | | 0.55 | | ND | | ND | | 1.45 | | 0 | | ND**^e^** | | ND**^e^** | | ND**^e^** | | ND**^e^** | |
| **mPY ^a^** | | 0.54 ± 0.2 | | 0.13 ± 0.00 | | 0.10 ± 0.00 | | 0.12 ± 0.00 | | 0.23 ± 0.00 | | 0.00 ± 0.00 | | 0.06 ± 0.00 | | 1.84 | | 0 | | 2.73 | | 2.29 | | 0.38 ± 0.11 | | 6.93 ± 0.12 | |
| **mPY**  **0.1 M Na acetate** | | 4.40 ± 0.72 | | 0.26 ± 0.00 | | 1.06 ± 0.01 | | 0.20 ± 0.01 | | 0.86 ± 0.13 | | 0.00 ± 0.00 | | 0.10 ± 0.00 | | 0.98 | | 0 | | 4.89 | | 5.64 | | 0.83 ± 0.46 | | 7.16 ± 0.01 | |
| **mPYF ^b^** | | 0.83 ± 0.01 | | 0.08 ± 0.02 | | 0.30 ± 0.01 | | 0.04 ± 0.0 | | 0.88 ± 0.05 | | 0.12 ± 0.01 | | 0.6 ± 0.00 | | 2.85 | | 5.06 ± 1.47 | | 21.98 | | 36.32 | | 2.90 ± 0.21 | | 6.17 ± 0.06 | |
| **mPYF**  **0.1 M Na acetate** | | 1.46 ± 0.01 | | 0.13 ± 0.02 | | 0.87 ± 0.10 | | 0.19 ± 0.23 | | 4.37 ± 0.00 | | 0.05 ± 0.00 | | 0.64 ± 0.00 | | 1.84 | | 11.12 ± 1.37 | | 16.04 | | 81.02 | | 5.45 ± 0.57 | | 6.48 ± 0.11 | |
| **mPYF**  **0.1M Na butyrate** | | 1.69 ± 0.01 | | 0.96 ± 1.12 | | 1.57 ± 0.08 | | 0.20 ± 0.00 | | 6.34 ± 0.00 | | 0.00 ± 0.00 | | 0.57 ± 0.005 | | 2.52 | | 10.10 ± 0.19 | | 17.21 | | 52.23 | | 3.90 ± 0.14 | | 6.34 ± 0.01 | |
| **mPYF**  **0.1 M Na acetate, 0.1 M Na butyrate** | | 4.70 ± 0.14 | | 0.16 ± 0.00 | | 2.50 ± 0.11 | | 0.25 ± 0.01 | | 9.72 ± 0.17 | | 0.00 ± 0.00 | | 0.64 ± 0.02 | | 3.96 | | 11.77 ± 0.98 | | 14.12 | | 57.67 | | 6.18 ± 0.49 | | 6.69 ± 0.01 | |
| **mPYF**  **0.1 M Na propionate** | | 1.35 ± 0.04 | | 2.28 ± 0.41 | | 0.21 ± 0.01 | | 4.06 ± 0.25 | | 0.18 ± 0.02 | | 2.03 ± 0.12 | | 0.11 ± 0.00 | | 2.83 | | 9.67 ± 0.66 | | 16.38 | | 54.41 | | 5.35 ± 0.32 | | 6.31 ± 0.02 | |
| **mPYF**  **0.1 M Na acetate,**  **0.1 M Na propionate** | | 4.47 ± 0.04 | | 1.92 ± 0.04 | | 1.01 ± 0.03 | | 5.74 ± 0.05 | | 1.47 ± 0.07 | | 2.71 ± 0.07 | | 0.20 ± 0.01 | | 4.23 | | 12.18 ± 1.44 | | 10.71 | | 76.74 | | 6.38 ± 0.39 | | 6.48 ± 0.06 | |
| **mPYF**  **0.1 M Na valerate** | | 1.13 ± 0.03 | | 0.21 ± 0.001 | | 0.08 ± 0.01 | | 6.33 ± 0.1 | | 0.14 ± 0.04 | | 2.39 ± 0.06 | | 0.12 ± 0.004 | | 0.22 | | 2.19 ± 0.26 | | 7.99 | | 17.56 | | 0.85 ± 0.07 | | 6.18 ± 0.01 | |
| **mPYF**  **0.1 M Na acetate,**  **0.1 M Na valerate** | | 4.92 ± 0.25 | | 0.65 ± 0.08 | | 0.15 ± 0.001 | | 5.78 ± 0.106 | | 0.00 ± 0.00 | | 3.56 ± 0.14 | | 0.16 ± 0.006 | | -0.87 | | 6.16 ± 0.14 | | 7.7 | | 29.58 | | 2.20 ± 0.71 | | 6.34 ± 0.02 | |
| **mPYF**  **0.1 M**  **Na caproate** | | 0.93 ± 0.04 | | 0.07 ± 0 | | 0.17 ± 0.0004 | | 0.00 ± 0.00 | | 9.27 ± 0.46 | | 0.00 ± 0.00 | | 0.15 ± 0.01 | | -0.99 | | 9.78 ± 5.0 | | 0.44 | | 1.53 | | 0.13 ± 0.11 | | 6.15 ± 0.02 | |
| **mPYF**  **0.1 M Na acetate,**  **0.1 M Na caproate** | | 6.39 ± 0.04 | | 0.09 ± 0.006 | | 0.17 ± 0.005 | | 0.00 ± 0.00 | | 9.68 ± 0.288 | | 0.00 ± 0.00 | | 0.18 ± 0.006 | | -0.97 | | 6.88 ± 2.80 | | 0.98 | | 2.09 | | 0.20 ± 0 | | 6.23 ± 0.03 | |
| **mPYF**  **0.1 M Na acetate,**  **0.05 M Na valerate** | | 5.13 ± 0.01 | | 0.47 ± 0.00 | | 1.17 ± 0.00 | | 5.18 ± 0.00 | | 1.96 ± 0.00 | | 3.21 ± 0.11 | | 0.00 ± 0.00 | | 6.12 | | ND | | ND | | ND | | ND | | ND | |
| **mPYF**  **0.1 M Na acetate,**  **0.05 M Na caproate** | | 4.97 ± 0.00 | | 0.15 ± 0.00 | | 1.57 ± 0.00 | | 0.24 ± 0.00 | | 8.7 ± 0.00 | | 0.00 ± 0.00 | | 1.15 ± 0.03 | | 4.78 | | ND | | ND | | ND | | ND | | ND | |

^a^ mPY: peptone yeast extract medium modified from DSMZ 104 medium (modified peptone yeast extract glucose medium)

^b^mPYF: peptone yeast extract fructose medium modified from DSMZ 104 medium

^c^ RCM: reinforced Clostridia medium used to isolate *M. hexanoica*

^d^Increase/decrease in amount of carboxylates

^e^ND: Not detected

**Optimization of Culture Conditions for *n*-Caproate Production by *M. hexanoica* using Response Surface Methodology**

The experimental protocol for testing various acetate and *n*-butyrate concentrations and pH values was set using the Design-Expert^®^ software of Stat-Ease (**Table S2**). Six experiments (numbers 15–20) were used as control conditions for statistical analysis. The first factor, A, was sodium acetate, and its probability was <0.02 in the analysis of variance (ANOVA), indicating that it was a significant independent factor in this model (**Table S3**). Factors B and C were sodium butyrate and pH, respectively, with values >0.05 and were, thus, not significant factors. The interaction factor AB was <0.0001, indicating that it was the most critical factor (**Table S3)**. Therefore, we hypothesized that the interaction between acetate and *n*-butyrate would increase *n*-caproate production. The medium was supplemented with 8.3 g/L acetate and 13.6 g/L *n*-butyrate; the predicted maximum *n*-caproate concentration was 10.61 g/L (**Figure S2**). This prediction was validated experimentally (**Figure S3**). The *n*-caproate yield was 11 g/L, which was too high to allow growth. Therefore, we hypothesized that the cellular toxicity of *n*-caproate may limit further increases in the *n*-caproate concentration. Hence, an extractive fermentation was performed (**Figure S5** and S**6**).

**Table S2.** Experimental design and results for *n*-caproate production using response surface methodology

| **Order** | **Sodium acetate** | | **Sodium butyrate** | **pH** | ***n-*Caproate** |
| --- | --- | --- | --- | --- | --- |
| 1 | -1 | -1 | | -1 | 6.5 |
| 2 | 1 | -1 | | -1 | 7.9 |
| 3 | -1 | 1 | | -1 | 9.7 |
| 4 | 1 | 1 | | -1 | 4.4 |
| 5 | -1 | -1 | | 1 | 6.0 |
| 6 | 1 | -1 | | 1 | 7.5 |
| 7 | -1 | 1 | | 1 | 10.4 |
| 8 | 1 | 1 | | 1 | 2.1 |
| 9 | -1.7 | 0 | | 0 | 6.9 |
| 10 | 1.7 | 0 | | 0 | 7.3 |
| 11 | 0 | -1.7 | | 0 | 5.6 |
| 12 | 0 | 1.7 | | 0 | 5.2 |
| 13 | 0 | 0 | | -1.7 | 8.2 |
| 14 | 0 | 0 | | 1.7 | 9.2 |
| 15 | 0 | 0 | | 0 | 10.8 |
| 16 | 0 | 0 | | 0 | 9.5 |
| 17 | 0 | 0 | | 0 | 10.4 |
| 18 | 0 | 0 | | 0 | 11.1 |
| 19 | 0 | 0 | | 0 | 10.0 |
| 20 | 0 | 0 | | 0 | 10.8 |

**Table S3.** ANOVA results for the optimum *n*-caproate production using response surface methodology

| **Source** | **F value** | **Prob > F** |  |
| --- | --- | --- | --- |
| Model | 14.69 | <0.0001 | significant |
| A | 7.18 | 0.0200 |  |
| B | 0.30 | 0.5929 |  |
| C | 0.05 | 0.8201 |  |
| AC | 0.87 | 0.3704 |  |
| BC | 0.05 | 0.8143 |  |
| AB | 33.43 | <0.0001 |  |
| Residual |  |  |  |
| Lack of Fit | 4.47 | 0.0592 | not significant |

**Quadratic Model**

***n*-Caproate** =10.43 – 0.73A – 0.15B – 0.063C – 1.19A^2^ – 1.79B^2^ – 0.61C^2^ – 2.06AB

R^2^: 0.8955

Adj R^2^: 0.8346

**a b**


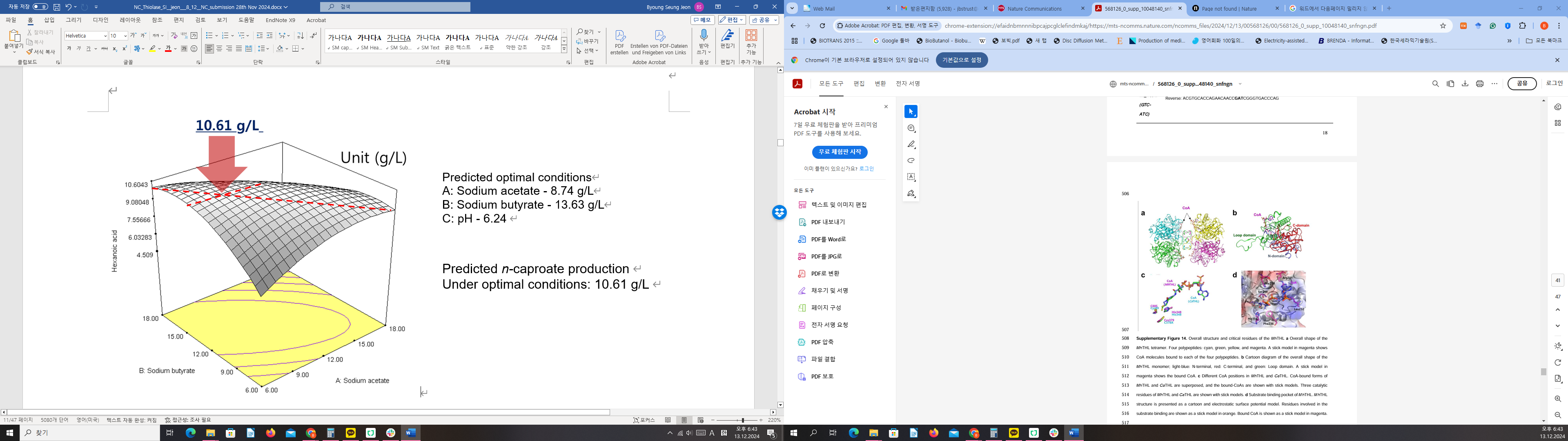

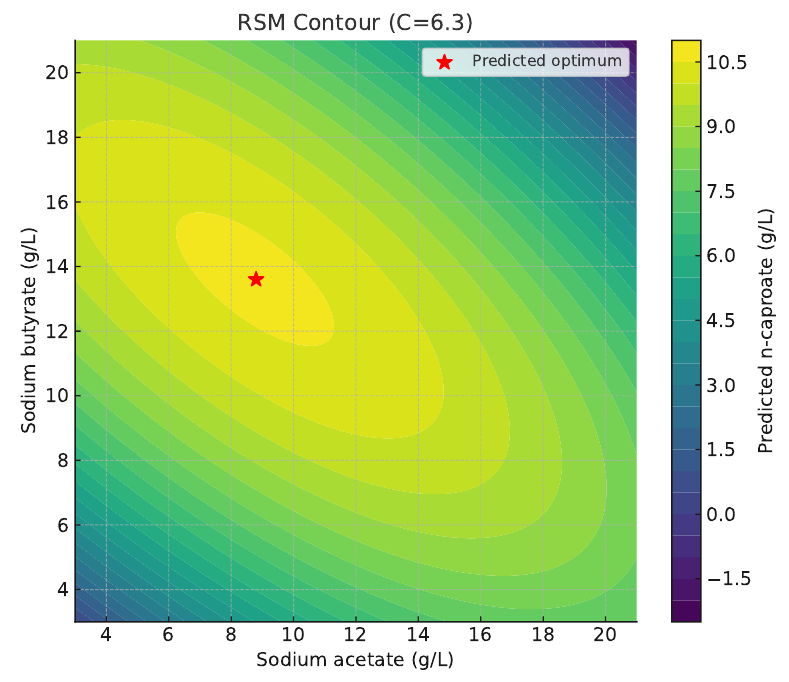


Predicted *n-*caproate

Under optimal conditions: 10.61 g/L

Predicted optimal conditions

A: Sodium acetate - 8.74 g/L

B: Sodium butyrate - 13.63 g/L

C: pH - 6.24

**c**


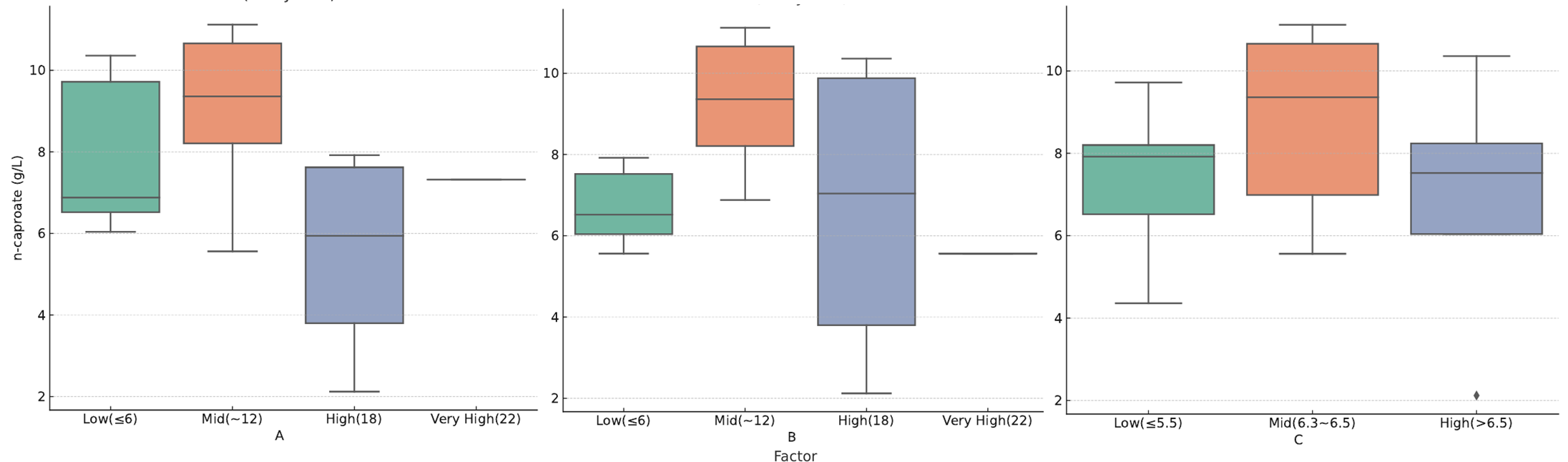


**Figure S2.** **a** Three-dimensional plot from RSM results. **b** RSM 2-dimensional contour plot from the RSM results. Experimental data were obtained from 20 runs (n = 20). Six runs at the central point were included to evaluate the significance of the model, and 14 runs were varied according to three different factors. Statistical analysis was performed using a quadratic response surface model (RSM) based on a central composite design (CCD) in Design-Expert® (Stat-Ease Inc., Minneapolis, MN, USA). All data in Figure S2 were analyzed by analysis of variance (ANOVA). The quadratic model predicted a maximum *n-*caproate production of 10.61 g/L under specific conditions. The evaluated factors were sodium acetate (A), *n-*butyrate (B), and pH (C). The optimal condition was 8.74 g/L sodium acetate, 13.63 g/L *n*-butyrate, and pH 6.24. When sodium acetate (factor A) and *n-*butyrate (factor B) are added independently, each factor is less significant than when added together. The interaction between A and B factors is quantified in Table S3; the probability of AB was <0.0001. **c** Post-hoc analysis of *n*-Caproate production under different factor levels. Boxplots show the distribution of *n*-Caproate concentrations (g/L) for each categorical level of Factor A (sodium acetate), Factor B (sodium butyrate), and Factor C (initial pH). Post-hoc comparisons were conducted using Tukey’s Honest Significant Difference (HSD) test. No statistically significant differences (p > 0.05) were detected among groups; therefore, group letters are not shown. Figures were generated using Python 3.11 (pandas, statsmodels, seaborn, and matplotlib).

**Figure S3.** Validation test of the condition predicted by Design-Expert^®^ to cause the maximum *n*-caproate production for *M. hexanoica.* All samples in **Figure S3** were measured in technical duplicate. Data are presented as the mean ± standard deviation, calculated using the Origin software.

**Evidence of the Carbon Chain Elongation Mechanism using ^13^C-labeled *n*-Butyrate**

As a preculture, *M. hexanoica* was cultured in mPYF medium containing 1% fructose. The mPYF medium supplemented with 0.05 M ^13^C labeled *n*-butyrate was inoculated with pre-culture (1% v/v) and incubated for 24 h at 37 °C. Subsequently, the mass patterns of the metabolic products were analyzed using GC-ToFMS, revealing that carbon positions 1 and 2 of the produced *n*-caproate were ^12^C-labeled. In contrast, ^13^C-labeled *n*-butyrate was located at carbon positions 3, 4, 5, and 6. Hence, the reaction pattern was identical to that catalyzed by *β-*ketothiolase, which catalyzes the condensation reaction between two acetyl-CoA molecules.

**a**


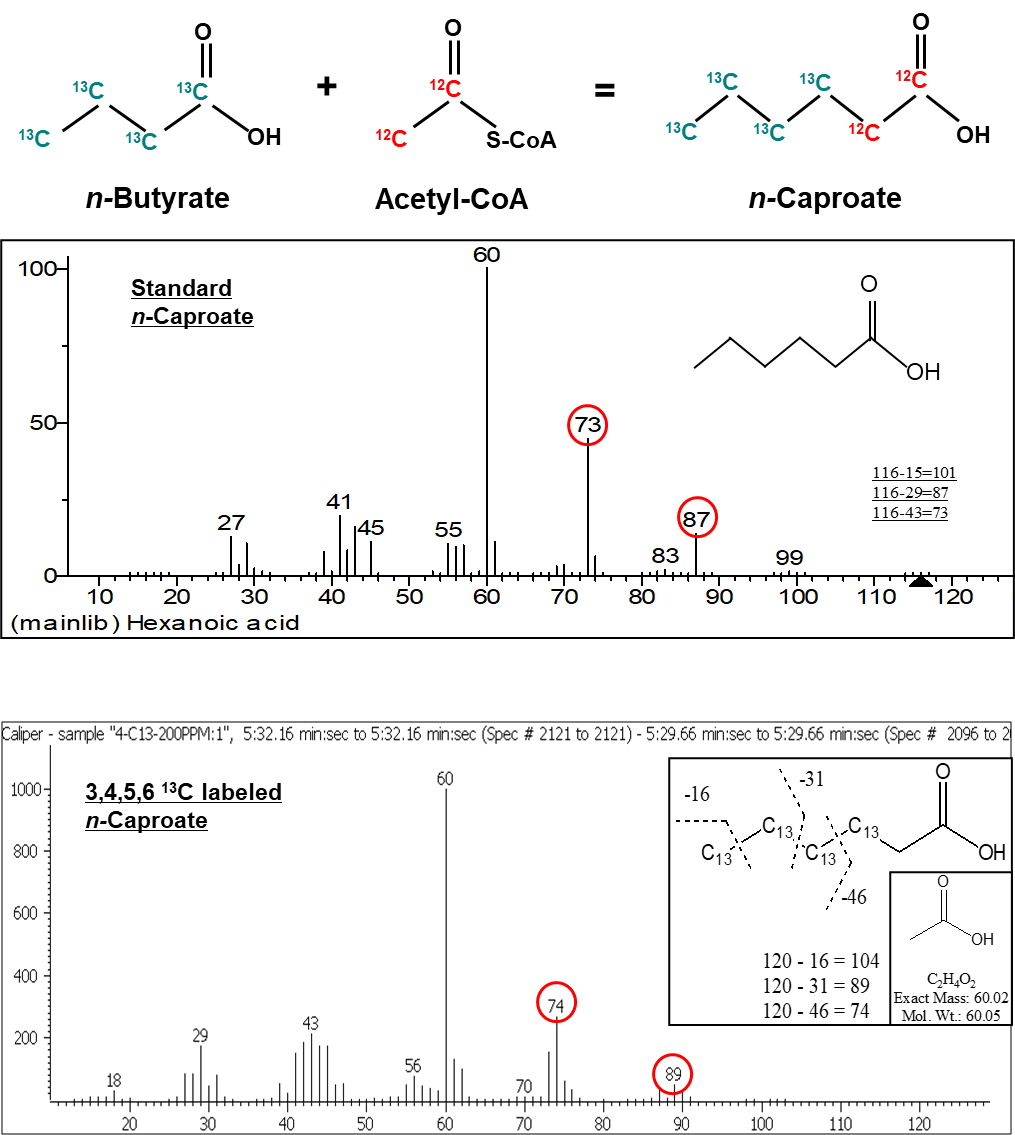


**c**

**b**

**Figure S4.** Mass spectrum of *n*-caproate produced using fully ^13^C-labeled *n*-butyrate. **a** Postulated molecular mechanism for *n*-caproate synthesis integrated with the ^13^C isotope. **b** Mass spectrum of standard *n*-caproate. **c** Mass spectrum of partially ^13^C labeled *n*-caproate synthesized by *M. hexanoica*. Ion masses of 73 and 87 were observed as a crucial fraction in standard *n*-caproate. Ion masses of 74 and 89 were observed in partially ^13^C-labeled *n*-caproate. In this study, *M. hexanoica* elongated extracellular ^13^C-labeled *n-*butyrate, which had a regular 3,4,5,6 ^13^C pattern in *n*-caproate. Identification of compounds was performed by comparing the mass spectra with the NIST library. Similarity scores and probabilities were calculated based on dot-product matching algorithms provided by the NIST library.

**Continuous *n-*Caproate Production by *M. hexanoica* using *In Situ* Extractive Fermentation**

The *in situ* extractive fermentation was conducted in a continuously stirred tank reactor. A mixture of oleyl alcohol and Alamine 336 was used as the extraction solvent, and its volume was double that of the fermentation broth (0.7 L). Extractive fermentation was continued until *n*-caproate was no longer produced. Nutrient and concentrated fructose sources were added whenever the fructose concentration in the fermentation broth dropped below 5 g/L. Meanwhile, the *n*-butyrate was injected according to the increase in pH and the pH was titrated to between 5.9 and 6.0. The *n*-caproate can be extracted at a higher pH than *n*-butyrate; therefore, the added butyrate was retained in the fermentation broth, whereas *n*-caproate was extracted at pH 5.9. *n*-Caproate was continuously produced in the solvent up to a concentration of ~130 g/L. Production was stopped when the *n*-caproate concentration increased to ~10 g/L in the broth phase, close to the maximum *n*-caproate concentration predicted by RSM. Thus, 10 g/L was expected to inhibit cell growth; therefore, it was expected that the production of *n*-caproate would continue via back extraction or by changing the fresh solvent. The OD_600_ decreased when concentrated nutrients and fructose were spiked into the reactor. Therefore, they are suspected of inducing cell damage, resulting in slower productivity. To circumvent this problem, concentrated nutrients and fructose sources were continuously injected using a syringe pump when the fructose content of the fermentation broth was below 5 g/L. The highest productivity achieved was 2 g/L/h, and 70 g of *n*-caproate was produced within two days (**Table S4**).


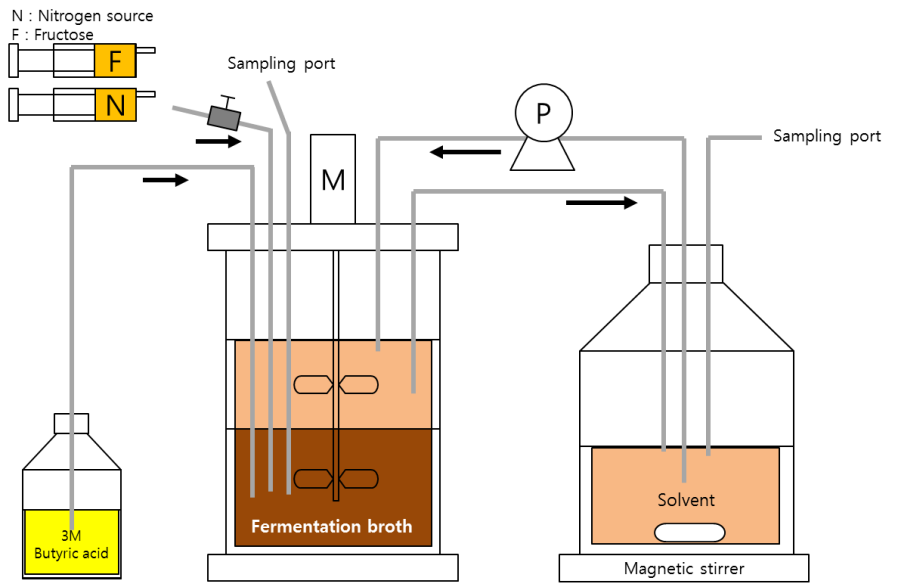


**a**

**F**: Fructose source

**N**: Nitrogen source

F and N were re-injected by a syringe pump when F in fermentation broth decreased to 5 g/L below.

**P**: Peristaltic Pump

**M**: Agitating motor

**Fermentation broth**: mPYF

**Solvent**: Alamine 336+oleyl alcohol

Solvent was circulated at 1 L/h.


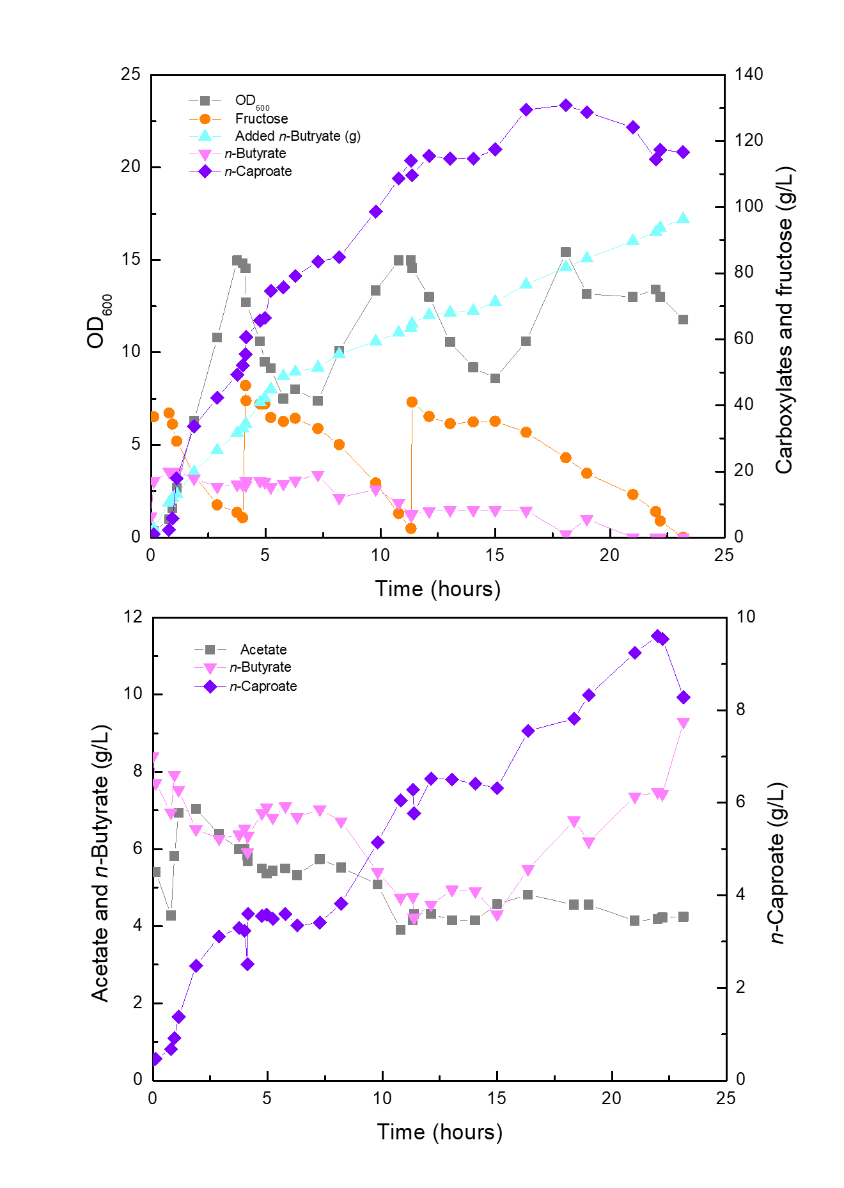


**b**

**c**

**Figure S5.** *n*-Caproate production using a fed-batch reactor and *in-situ* extraction. **a** Schematic diagram of the extractive fermentation process. **b** *n*-Caproate was found in the solvent layer; fructose and *n*-butyrate were added; cell growth occurred in the medium layer. **c** Acetate, *n*-butyrate, and *n*-caproate were found in the solvent layer; solvent volume: 1.4 L, fermentation broth initial volume: 0.7 L, final volume: 1 L. The reactor was operated in continuous mode; independent experimental replicates could not be performed.


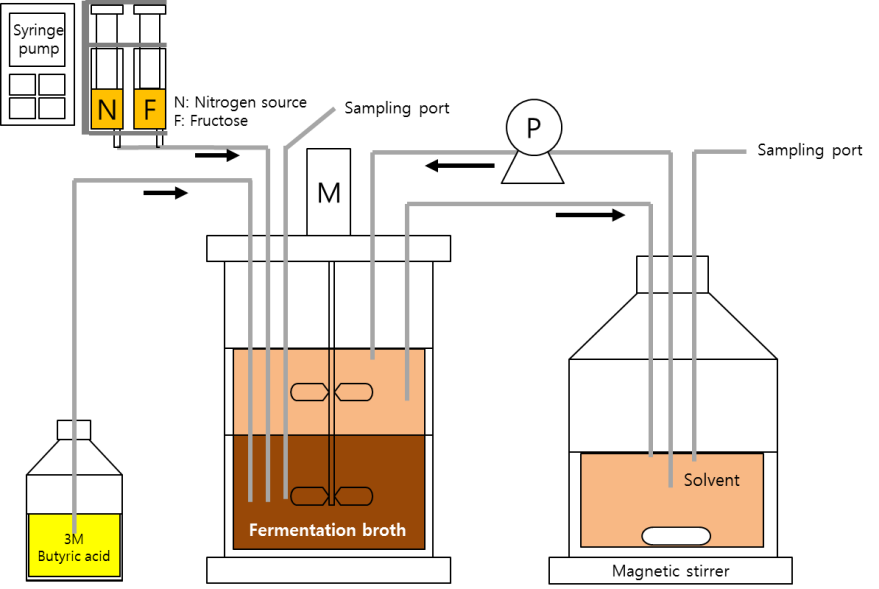


pH CTRL

Sensor

**a**

**F**: Concentrated fructose

**N**: Nitrogen source

F and N were supplied between 0.5 and 1.5 g/L/h by syringe pump. The speed was determined by the consumption rate.

**P**: Peristaltic pump

**M**: Agitating motor

**Fermentation broth**: mPYF mentioned in manuscript

**Solvent**: Alamine 336 + oleyl alcohol; Solvent was circulated at 1 L/h


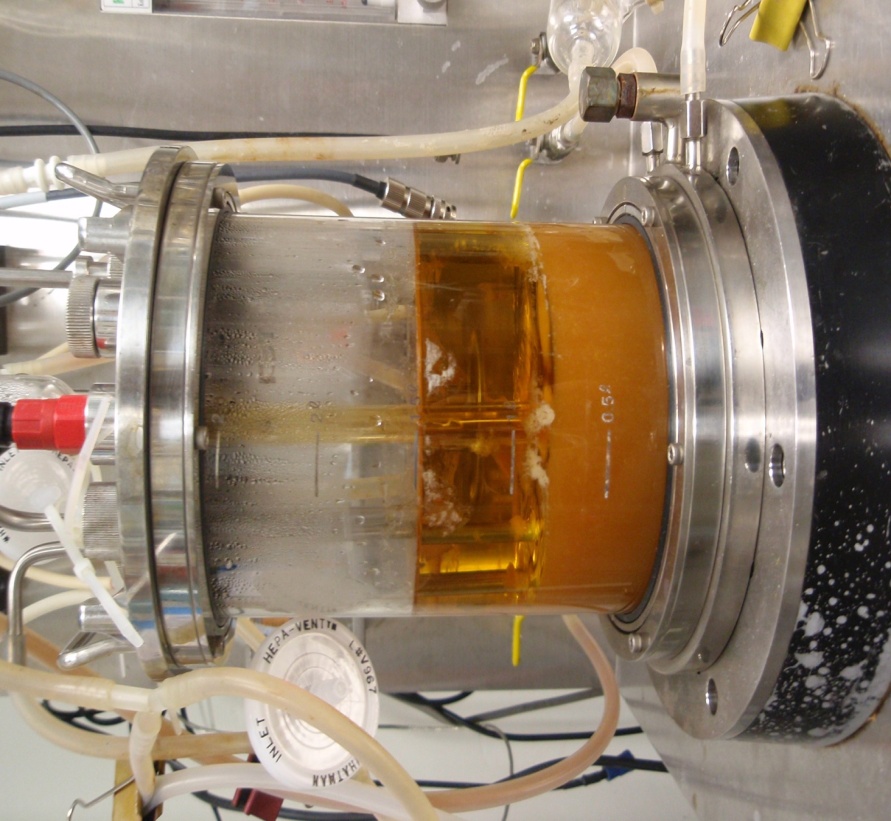


**Medium 700 ml**

**Solvent 700 ml**

**Solvent**

**Oleyl alcohol (9)**

**Alamine 336 (1)**

**100 RPM, Temp 40 C**

**b**

**Figure S6.** *n-*Caproate production using a semi-fed-batch reactor and *in-situ* extraction. **a** Schematic diagram of the extractive fermentation process. **b** Produced *n-*caproate amount (g) and *n-*added butyrate and fructose amount (g). The *n*-caproate was extracted into the solvent layer immediately, and the solvent was sampled to measure *n*-caproate. The solvent volume was 0.7 L and the initial and final volume of fermentation broth was approximately 0.7 L and 1 L, respectively. The produced *n-*caproate was 70 g/L. The fructose was supplied using a syringe pump, and the concentration was maintained at approximately 20 g/L in the fermentation broth. In total, 50 g fructose was added during overall fermentation; the added amount was measured using a scaled syringe. The cell growth (OD_600nm_) in the fermentation broth was measured at 600 nm using photo spectrometry. The consumed *n*-butyrate was measured using the scaled cylinder. The *n-*butyrate was injected by the dosing pump whenever pH was changed. The reactor was operated in continuous mode; independent experimental replicates could not be performed.

**Table S4**. Comparison of biological *n*-caproate production using two different operations

|  | pH | pH adjust regent | Added fructose | Added  *n*-butyric acid | Productivity | *Effective  *n*-caproic acid amount | Total carbon yield |
| --- | --- | --- | --- | --- | --- | --- | --- |
| **^†^Fed batch**  **(g)** | 5.99 | 3 M Butyrate | 108 g | ~95 g | 0.27 g/L/h | *182 g | 0.91 g CA /g carbon source |
| **^†^Fed batch**  **(mole)** | - | - | 0.6 mole | 1.08 mole | - | 1.56 mole | 0.92 mole CA / mole carbon source |
|  |  |  |  |  |  |  | ^&^Carbon selectivity of *n*-caproic acid: 89.14% |
| **‡ Semi-fed batch**  **(g)** | 5.99 | 3M Butyrate | 50 g | ~40 g | 2 g/L/h | *70 g | 0.77 g CA /g carbon source |
| **‡ Semi-fed batch**  **(mole)** | - | - | 0.28 mole | 0.45 mole | - | 0.63 mole | 0.86 mole CA / mole carbon source |

The effective *n*-caproate amount was calculated using the equation given below.

* Effective *n*-caproate amount = Solvent volume x concentration of *n*-caproate in solvent / Final fermentation volume (1L)

†Fed batch: Fed batch was operated with periodic injection of concentrated nutrient source (Total ~75 g) and carbon source (Fructose). Concentrated nutrient source (Total ~75 g) and carbon source (Fructose) were injected manually using a 50 ml disposable syringe.

‡Semi-fed batch: Semi-fed batch was operated with continuous injection of concentrated nutrient source (Total ~50 g) and fructose. A syringe pump was used for injecting concentrated nutrient sources and carbon sources.

^&^Carbon selectivity % = (*n*-Caproate mole (1.56) / *n*-Caproate mole (1.56) + *n*-butyrate mole (0.14) + *n*-acetate mole (0.05)) x 100. Therefore, carbon selectivity for *n*-caproate was 89.14 %.

The nutrient source was composed of yeast extract, tryptone, and their compositional ratio was 10:5:5:5. The nutrient source was prepared according to the protocol of DSMZ medium 104.

***M. hexanoica* Genome**

The genome of *M. hexanoica* is shown in **Figure 7**. Critical genes are marked on the Circos map. The thiolase of *M. hexanoica* was independently positioned in the middle of the genome. *crt-hbd*_2206-2207 were near each other. The *acdhs* used in this study were labeled *bcdh*_2230 and *acdh*_2251. Eight *act* genes were broadly positioned in the genome. *M. elsdenii* was the closest relative to *M. hexanoica.* However, the genome of *M. hexanoica* is larger. The chromosome size of *M. hexanoica* was 2,877,851 bp and *M. elsdenii* was 2,474,718 bp (**Figure S7**) and encodes more genes related to the reverse β-oxidation pathway (**Figure S7** and **Table S5**). Furthermore, *M. hexanoica* encodes only one thiolase, whereas *M. elsdenii* encodes two thiolases. The rBOX genes of *M. hexanoica* were compared with those of *Clostridium acetobutylicum,* a well-known butanoate producer, and *M. elsdenii,* an *n*-caproate producer, in **Table S5**. *M. hexanoica* differs from *M. elsdenii* at the genus level and the taxon of *M. hexanoica* may need to be reestablished in the future.


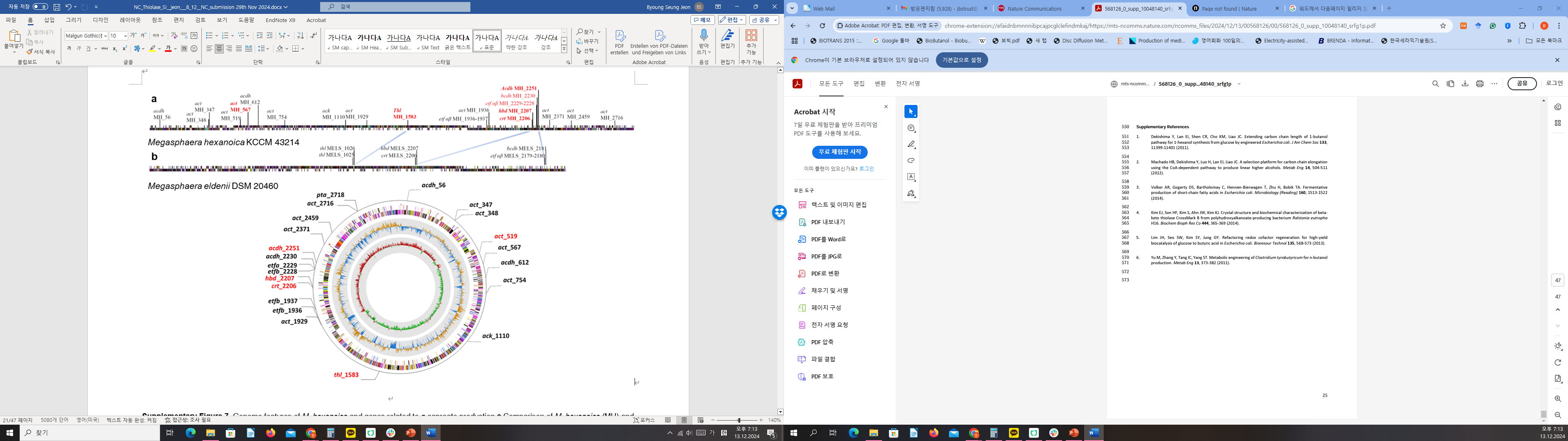


**Figure S7.** Genome features of *M. hexanoica* and genes related to *n*-caproate production **a** Comparison of *M. hexanoica* (MH) and *M. elsdenii* (MEL) at the genomic level; **b** Circos map of *M. hexanoica.* Figures were generated with CLgenomics software

**Table S5.** Comparison of orthologous genes with the closest neighbors

|  | ***thl*** | ***hbd*** | ***Crt*** | ***acdh*** | ***act*** | ***Ctf* αb** |
| --- | --- | --- | --- | --- | --- | --- |
| *Megasphaera hexanoica* | *thl*_1583 | *hbd*_2207  *hbd*_2534 | *crt*_2206  *crt*_613 | *acdh*_56, *acdh*_612, *acdh*_2230, and *acdh*_2251 | *act*_348, *act*_347, *act*_567, *act*_519  *act*_754, *act*_1929, *act*_2371, and *act* _2459 | None |
| *Megasphaera elsdenii*  DSM20460 | *MELS*_1025 and 1026 | *MELS*_1448 | *MELS*_1449 and 462 | *MELS*_63, *MELS*_461, *MELS*_747, *MELS*_1181, *MELS*_2128 | *MELS*_341, *MELS*_415, *MELS*_430, and *MELS*_437 | None |
| *Clostridium acetobutylicum*  ATCC824 | *CA*_C2873 *CA*_P0078 | *CA*_C2708 | *CA*_C2712 | *CA*_C2711 | None | *CA*_P0163-0164 |

**RNA Transcriptomic Analysis**

RNA transcriptome analysis was performed based on the sequenced genome. Raw data were processed using CLRNA and normalized to relative log expression (RLE). The results are presented in **Figure S8, S9, and S10** and **Table S8.** Genes related to CoA were highly expressed (*thl*_1583, *hbd*_2207, and *crt*_2206). These genes were ranked in the top 15^th^ under all sampled conditions. Independent of the acyl-CoA dehydrogenases, *acdh*_2251 showed the highest rank. The expression of *acdh*_2251 was three-fold higher than that of *bcdh*_2230 (15^th^ highest). Of all genes in the *bcdh-etf* αβ cluster, *etf* α_2228 showed the highest value. *act_*567 exhibited the highest expression value among all *act* genes. *act*_567 likely plays an essential role in *n*-caproate production by transferring a CoA molecule from caproyl-CoA (hexanoyl-CoA) to *n*-butyrate or acetate.


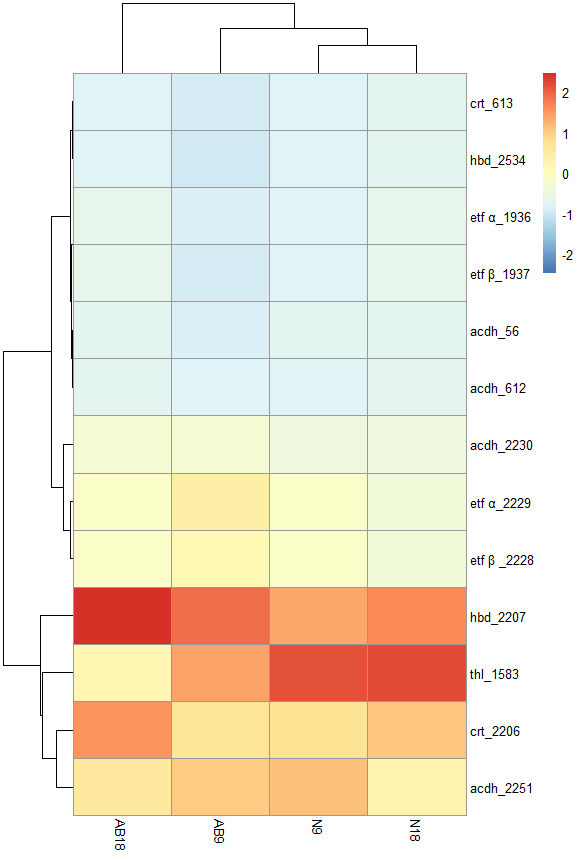


**a**

**b**


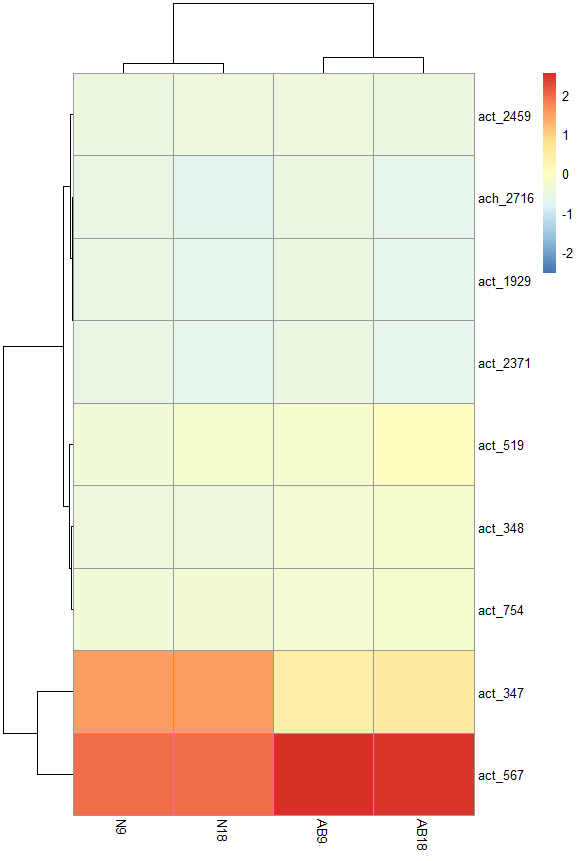


Acetate CoA-transferases Core genes related to r-BOX

**Figure S8.** Transcription patterns of genes related to reverse beta oxidation (r-BOX) in *M. Hexanoica* under four different conditions. The expression levels between genes of each sample were normalized using the RLE. **A** Transcription patterns for core genes related to acetate CoA-transferases (RNA expression range: 19.44–7816.49 RLE). **B** Transcription patterns for core genes of r-BOX (RNA expression range: 74842–651 RLE). AB9: Conditions for production of *n*-caproate in the exponential growth phase (9 h). N9: Conditions for no production of *n*-caproate in the exponential growth phase (9 h). AB18: Conditions for production of *n*-caproate in the stationary growth phase (18 h). N18: Conditions for no production of *n*-caproate in the stationary growth phase (18 h). RNA-seq read counts from four samples were normalized using reads per kilobase per million mapped reads (RPKM), trimmed mean of M-values (TMM), and relative log expression (RLE) normalization. For the heat map shown in **Figure S8**, relative log expression (RLE) normalization was used, as it better accounts for differences in library size and enables more accurate comparison of gene expression levels across all samples.

**Evaluation of ACTs Derived from *M. Hexanoica* using Genetically Modified *Escherichia coli***

The transformed *E. Coli* platform strains produced the corresponding alcohols based on the carbon number of the added carboxylates (*i.e.*, propionate: propanol, *n*-butyrate: *n*-butanol, *n*-valerate: *n*-pentanol, *n*-caproate: *n*-hexanol).


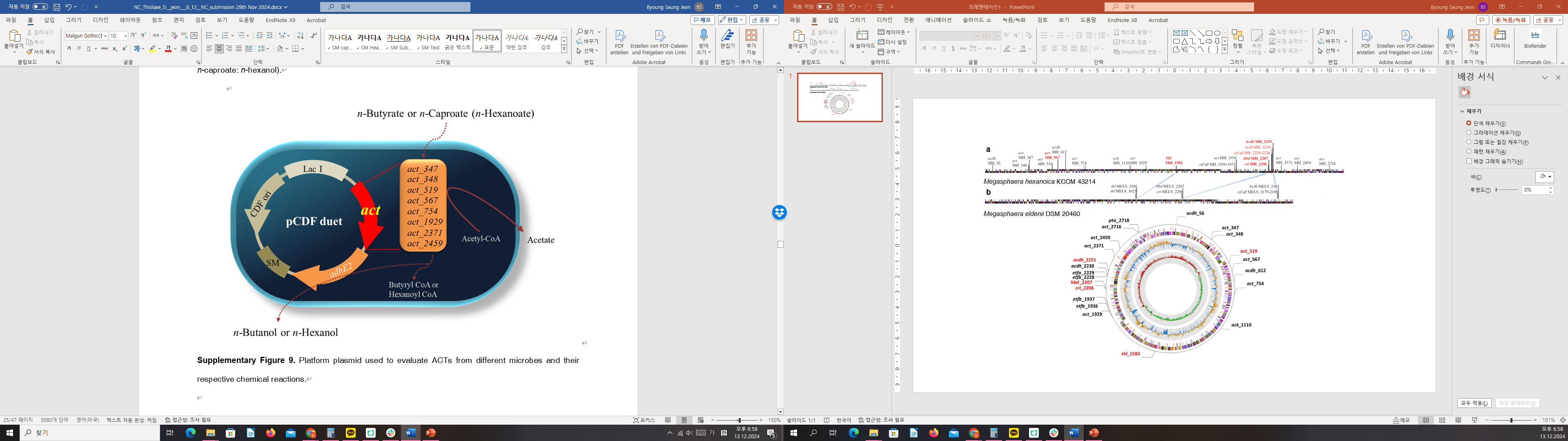


**Figure S9.** Platform plasmid used to evaluate ACTs from different microbes and their respective chemical reactions.

**Table S6.** List of *Escherichia coli* platform strains used to evaluate different ACTs

| **Name** | **Relative characteristics** | **source** |
| --- | --- | --- |
| **Strains** |  |  |
| *Clostridium acetobutylicum* |  | ATCC 824 |
| *E. coli* DSM01 | MG1655(DE3)∆*frdA*::FRT∆*pta*::FRT∆*ldhA*::FRT∆*adhE*::FRT | Baek et al. (2013) |
| *E. coli* DH5α | F-80dlacZ ∆M15 ∆(lacZYA-argF) U169 recA1 endA1 hsdR17(r_k_^-^,m_k_^+^) phoAsupE44-thi-1 gyrA96 relA1 | BIOFACT |
| E. coli pCDF | DH5α/pCDFDuet-1 | This work |
| *E. coli* ACT#1 | DSM01/pCDF-A1 | This work |
| *E. coli* ACT#2 | DSM01/pCDF-A2 | This work |
| *E. coli* ACT#3 | DSM01/pCDF-A3 | This work |
| *E. coli* ACT#4 | DSM01/pCDF-A4 | This work |
| *E. coli* ACT#5 | DSM01/pCDF-A5 | This work |
| *E. coli* ACT#6 | DSM01/pCDF-A6 | This work |
| *E. coli* ACT#7 | DSM01/pCDF-A7 | This work |
| *E. coli* ACT#8 | DSM01/pCDF-A8 | This work |
| *E. coli* ACT#9 | DSM01/pCDF-A9 | This work |
| **Plasmids** |  |  |
| pCDFDuet-1 | Expression vector, Sm^R^, CDF ori | Novagen (EMD Millipore) |
| pCDF-adhE2 | pCDFDuet-1, but P_lac_::atoB-adhE2 | This work |
| pCDF-A1 | pCDFDuet-1, but P_lac_::atoB-adhE2-ACT 567 | This work |
| pCDF-A2 | pCDFDuet-1, but P_lac_::atoB-adhE2-ACT 347 | This work |
| pCDF-A3 | pCDFDuet-1, but P_lac_::atoB-adhE2-ACT 348 | This work |
| pCDF-A4 | pCDFDuet-1, but P_lac_::atoB-adhE2-ACT 2371 | This work |
| pCDF-A5 | pCDFDuet-1, but P_lac_::atoB-adhE2-ACT 1929 | This work |
| pCDF-A6 | pCDFDuet-1, but P_lac_::atoB-adhE2-ACT 754 | This work |
| pCDF-A7 | pCDFDuet-1, but P_lac_::atoB-adhE2-ACT 519 | This work |
| pCDF-A8 | pCDFDuet-1, but P_lac_::atoB-adhE2-ACT 2459 | This work |

**Table S7.** List of primers used for plasmid construction

| **Name** | **Sequence (5′–3′)** |
| --- | --- |
| ADHE2_F | TATATCATATGATGAAAGTTACAAATCAAAAAGAACTAAAA |
| ADHE2_R | TATATGAGCTCTTAAAATGATTTTATATAGATATCCTTAAGTT |
| ACT#1_F | TATATGGATCCCATGTACAAACTTTCACAAATTGCAG |
| ACT#1_R | TATATAAGCTTTTAGTATTCTGTCTTGCTCGTCT |
| ACT#2_F | TATATGGATCCCATGTCAGAATGGACGGATATGTA |
| ACT#2_R | TATATAAGCTTTTACCGTTTATTGCTCTTGCGC |
| ACT#3_F | TATATGAGCTCCATGGATGTAATGCAAGAATATGCC |
| ACT#3_R | TATATGTCGACTTAGATCAGGATATGCATTTTTTTAGC |
| ACT#4_F | TATATGAGCTCCATGACACAATATGAAACAATGTATGAA |
| ACT#4_R | TATATGTCGACTTACCAAATCATGTATTTGTCAACAG |
| ACT#5_F | TATATGGATCCCATGATGAATCAATGGCAGCGCAT |
| ACT#5_R | TATATAAGCTTTTACACAATAATATGCATCTTTCTGG |
| ACT#6_F | TATATGGATCCCATGGATTACCAGAGTGAATACATGA |
| ACT#6_R | TATATAAGCTTTTATCGTTTATGAGAAGCGCGC |
| ACT#7_F | TATATGGATCCCATGAATCCATTCGAAATATATCAGG |
| ACT#7_R | TATATAAGCTTTTATTTCTTGTTACTGTTCCTCCAG |
| ACT#8_F | TATATGAGCTCCATGATTGACATTTCAGATCGCATA |
| ACT#8_R | TATATGTCGACTTACTTCATACTCCCTGTTTCCA |


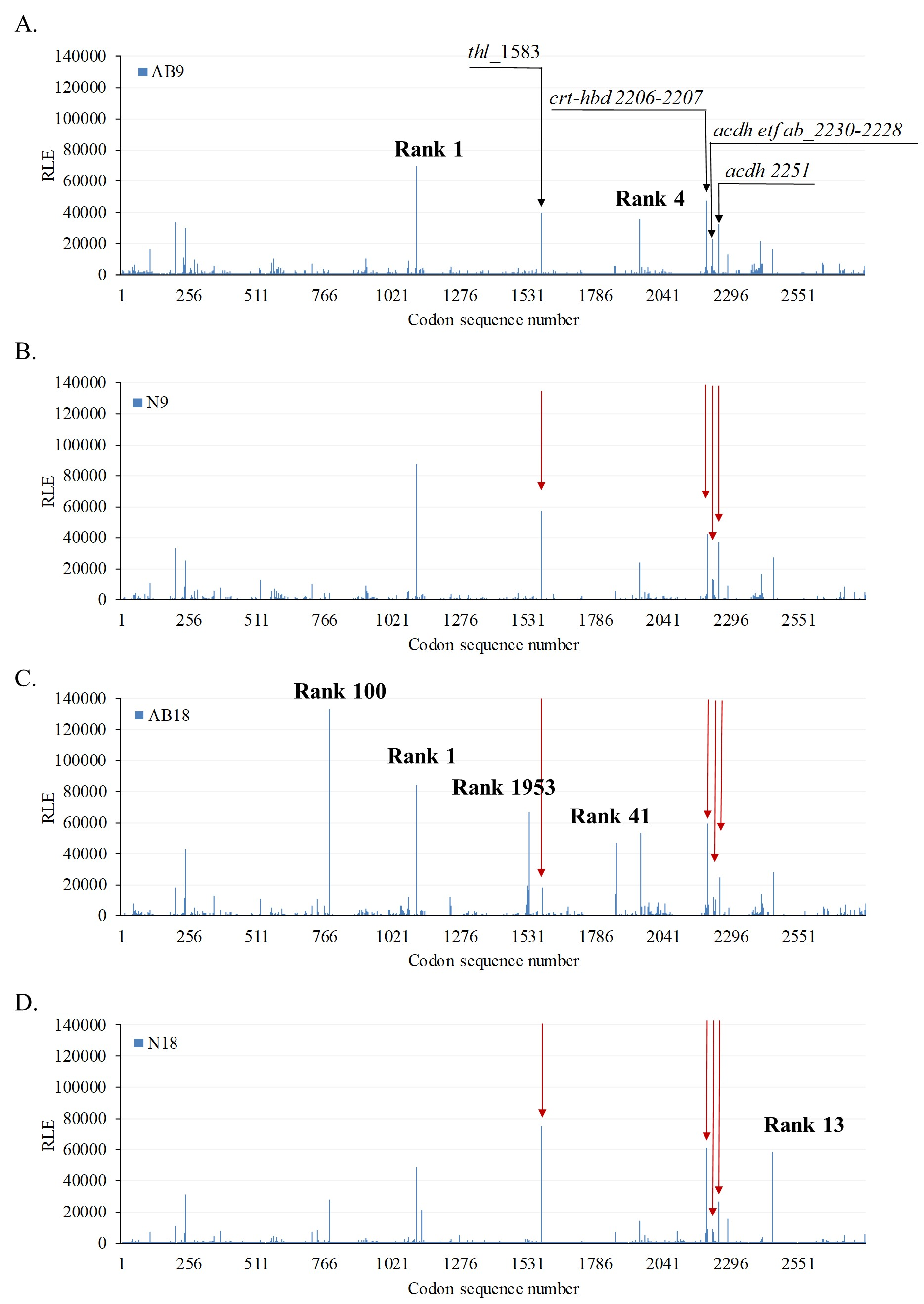


**a**

**b**

**c**

**d**

**Rank 13**

**Figure S10.** Relative log expression (RLE) values for RNA transcription of *M. Hexanoica* under the condition of producing *n*-caproate. Four conditions were applied for RNA transcriptome analysis: AB9, N9, AB18, and N18. AB: *n*-caproate-production condition; N: non-*n*-caproate-production condition; numbers following “AB” and “N” represent cultivation times (9: exponential phase, 18: stationary phase). For the four data shown in **Figure S10**, relative log expression (RLE) normalization was used.

**Table S8.** mRNA ranking under *n*-caproate-producing conditions at 9 h (AB9)

| **Rank** | **CDS** | **Gene** | **RLE** |
| --- | --- | --- | --- |
| 1 | 1113 | S-layer protein | 69855.32 |
| **2** | **2207** | **3-hydroxybutyryl-CoA dehydrogenase (*hbd*_2207)** | **47766.68** |
| **3** | **1583** | **Acetyl-CoA C-acetyltransferase (*thl*_1583)** | **40029.09** |
| 4 | 1953 | Adhesin Ata autotransporter | 35650.8 |
| 5 | 205 | Elongation factor Tu | 33790.36 |
| **6** | **2251** | **Acyl-CoA dehydrogenase (*acdh*_2251)** | **32850.28** |
| 7 | 243 | Glyceraldehyde 3-phosphate dehydrogenase | 29832.81 |
| 8 | 2206 | **Enoyl-CoA hydratase, Crotonase (*crt*_2207)** | 27538.49 |
| **9** | **2229** | **Electron transfer flavoprotein beta-subunit (*etf* β_2229)** | **22726.32** |
| 10 | 2410 | Elongation factor G | 21496.98 |
| **11** | **2228** | **Electron transfer flavoprotein alpha-subunit (*etf* α_2228)** | **18808.21** |
| 12 | 108 | Pyruvate-flavodoxin oxidoreductase | 16665.09 |
| 13 | 2455 | Belongs to the Glu/Leu/Phe/Val dehydrogenases | 16211.48  (AB18: 27812) |
| 14 | 2285 | The chaperonin (HSP60) family. 60 kDa chaperonin | 13009.95 |
| **15** | **2230** | **butyryl-CoA dehydrogenase, short-chain specific (*acdh*_2230)** | **11333.58** |
| 41 | 1862 | Methionine adenosyltransferase | 5752  (AB18: 46653) |
| 100 | 783 | O-acetylhomoserine aminocarboxypropyltransferase | 3234  (AB18: 133288) |
| 1953 | 1526 | Hypothetical protein | 46  (AB18: 19362) |

Bold characters indicate genes related to the reverse *β*-oxidation pathway.


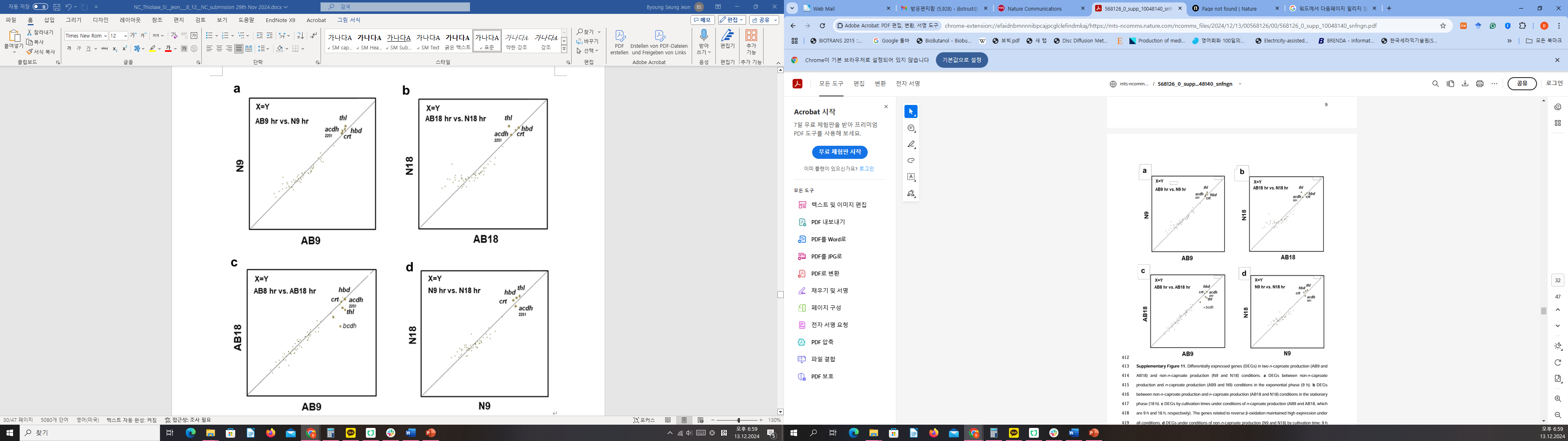


**Figure S11.** Differentially expressed genes (DEGs) in two *n*-caproate production (AB9 and AB18) and non-*n*-caproate production (N9 and N18) conditions. **A** DEGs between non-*n*-caproate production and *n*-caproate production (AB9 and N9) conditions in the exponential phase (9 h). **B** DEGs between non-*n*-caproate production and *n*-caproate production (AB18 and N18) conditions in the stationary phase (18 h). **C** DEGs by cultivation times under conditions of *n*-caproate production (AB9 and AB18, which are 9 h and 18 h, respectively). The genes related to reverse β-oxidation maintained high expression under all conditions. **D** DEGs under conditions of non-*n*-caproate production (N9 and N18) by cultivation time: 9 h (exponential phase) and 18 h (stationary phase). Differential expression analysis across the four conditions (AB9, AB18, N9, and N18) was performed using CLRNA software (ChunLab, Seoul, Korea). Statistical significance of gene expression differences was assessed within the software, and genes with adjusted p-values < 0.05 were considered differentially expressed.

**Construction of a Metabolic Pathway Producing *n*-Caproate using Genes Derived from *M. Hexanoica***

A metabolic pathway to produce *n*-caproate was constructed by inserting heterologous genes derived from *M. Hexanoica* into *E. Coli*; *thl, hbd,* and *crt* were selected based on genome and transcriptome analyses; *thl* from *M. Hexanoica* was inserted into the pCOLADuet-1 vector. Meanwhile, *hbd* and *crt* were cloned into the pCDFDuet-1 vector; *act_*567 was evaluated in the previous step and inserted into the pCDFDuet-1 vector. The evaluation using eight gene combinations was focused on identifying acyl-CoA dehydrogenase or butyryl CoA dehydrogenase of the reverse β-oxidation pathway. Trans-enoyl-CoA reductase from *T. Denticola* was used as a control.^[1]^ The constructed vectors were inserted into *E. coli* MG1655 DE3 (△ldh, △adhE, △frdA, and △pta). The amount of produced *n*-caproate was measured via GC-FID. Evaluation of eight gene sets revealed that *acdh*_2251 was associated with higher *n*-caproate production. The optimal *n*-caproate production combination was *hbd*_2207, *crt*_2206, *acdh*_2251, act_567, and *thl*_1583. All genes of the reverse β-oxidation pathway were derived from *M*. *hexanoica*. This combination was used to evaluate the activities of various thiolases.


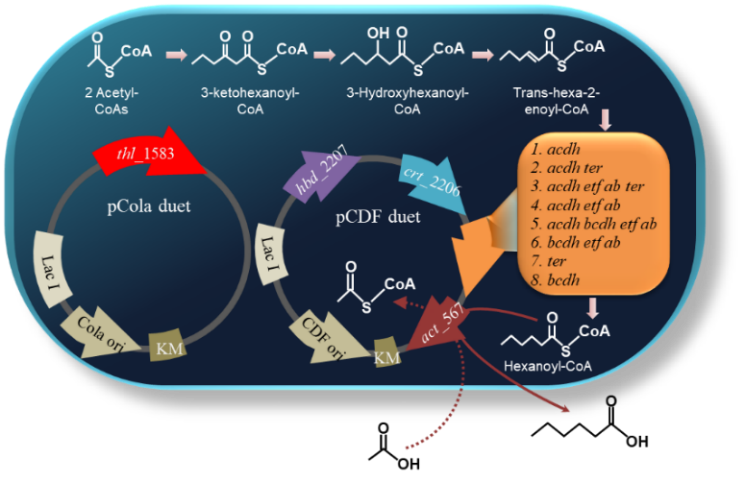

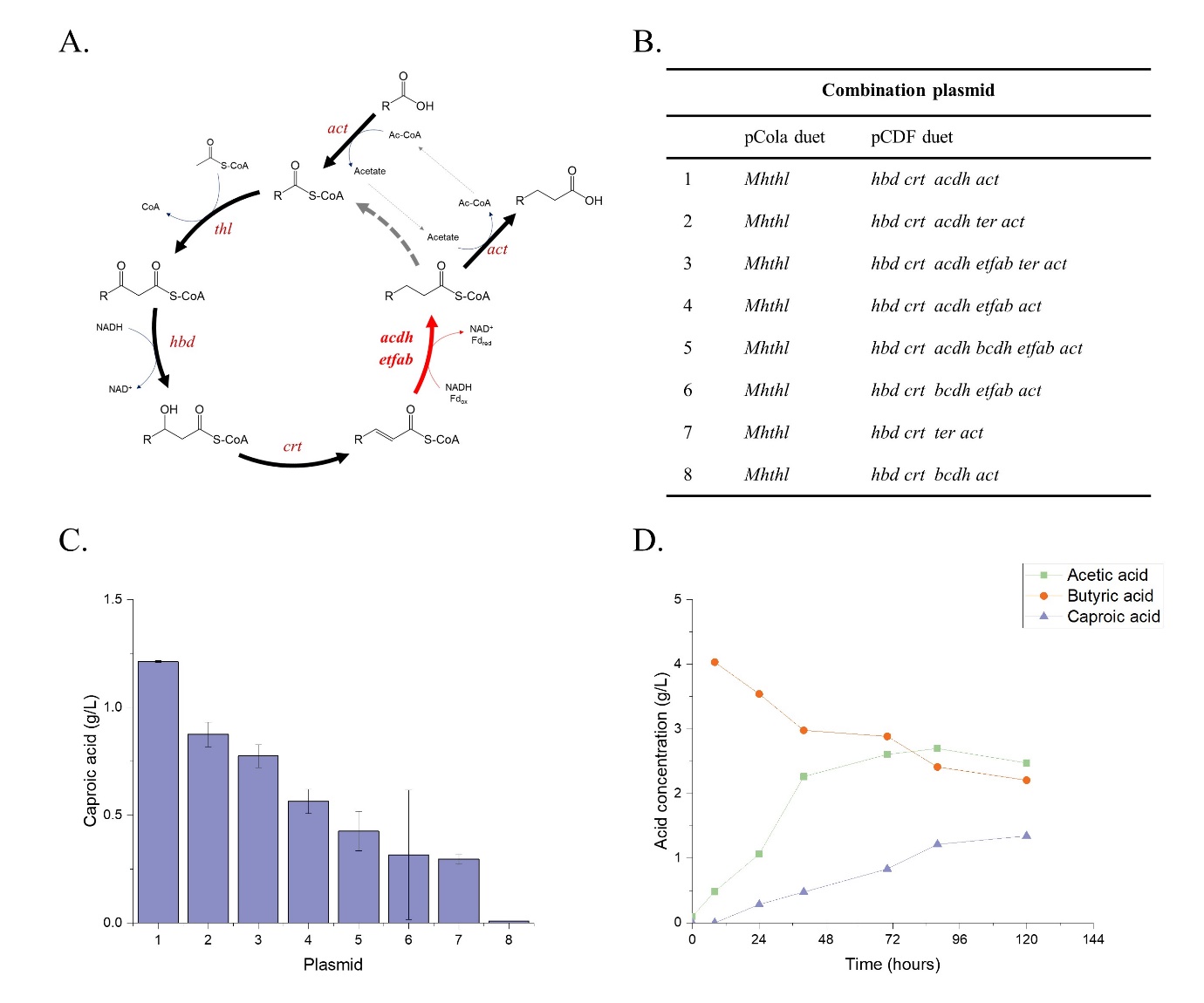


**Figure S12.** Plasmid construction and selected genes for *n*-caproate production in *E. coli*. The pCOLADuet-1 contained *thl* derived from *M. hexanoica*; *hbd, crt,* and *act* were inserted into pCDFDuet-1. Different combinations of *acdh*, *bcdh*, *ter*, and *etf* αβ were inserted into the vector between the *crt* and *act* genes. Both plasmids were inserted into *E. coli* MG1655 DE3 (△*ldh*, △*adhE*, △*frdA,* and △*pta*)

**Table S9.** Genes used to construct an *n-*caproate-producing pathway in *Escherichia coli*

| **Abbreviated name** | **Enzyme** | **Gene I.D. locus tag** |
| --- | --- | --- |
| **THL** | **Acetyl-CoA acetyltransferase** | ***thl*_1583** |
| **HBD** | **3-hydroxyacyl-CoA dehydrogenase** | ***hbd*_2207** |
| **CRT** | **Enoyl-CoA hydratase** | ***crt*_2206** |
| ACDH | Acyl-CoA dehydrogenase | *acdh*_2251 |
| BCDH | Butyryl-CoA dehydrogenase | *bcdh*_2230 |
| TER | Trans-enoyl-CoA reductase from *T. denticola* | 2741560^*^ |
| ETF α | Electron transfer flavoprotein | etf_α_2228 |
| ETF β | Electron transfer flavoprotein | etf_ β_2229 |
| **ACT** | **Acetate CoA-transferase** | ***act*_567** |

^*^NCBI Gene number

Bold characters represent platform genes to identify the primary genes responsible for the reverse β-oxidation pathway.

**Performance Evaluation of Five Thiolases for *n*-Caproate Production**

The five evaluated thiolases were *Mh*THL, *Ck*THL, *Re*BktB, *Ec*AtoB, and *Ca*THL. *Mh*THL is derived from *Megasphaera hexanoica* and produces MCCs from sugars. Mutations in the catalytic residues (L87V and V351I) of *Mh*THL were introduced to reduce the pocket size of the respective thiolase. *Ck*THL was obtained from *C. kluyveri* DSM 555 and produces *n*-caproate from ethanol and CKL_3696 ThlA1.^[2]^ *Re*BktB from *Ralstonia eutrohpa* H16 (*Cupriavidus necator*) is involved in the synthesis of *n*-butyrate (C_4_) and *n*-valerate (C_5_).^[3]^ *Ec*AtoB condenses two acetyl-CoA molecules in the *E. coli* platform to produce *n*-butyrate and is derived from the AtoDAEB cluster of *E. coli*.^[4]^ *Ca*THL is a thiolase from *Clostridium tyrobutyricum* ATCC 25755 that produces *n*-butyrate (C_4_).^[5]^ The respective *thl* genes were inserted into the first multiple cloning sites of the pCOLADuet-1 vector. pCOLADuet-1 vectors containing *thl* were transferred into *E. coli* MG1655 DE3(△*ldh*, △*adhE*, △f*rdA* and △*pta*), with the pCDFDuet-1 plasmid encoding *hbd*_2207, *crt*_2206, *acdh*_2251, and *act*_567. Strains were cultivated; 1 mM IPTG was added to induce protein expression, reaching 0.8 OD_600_. The *n*-caproate-producing performance of each strain was compared by quantifying concentrations using GC-FID.


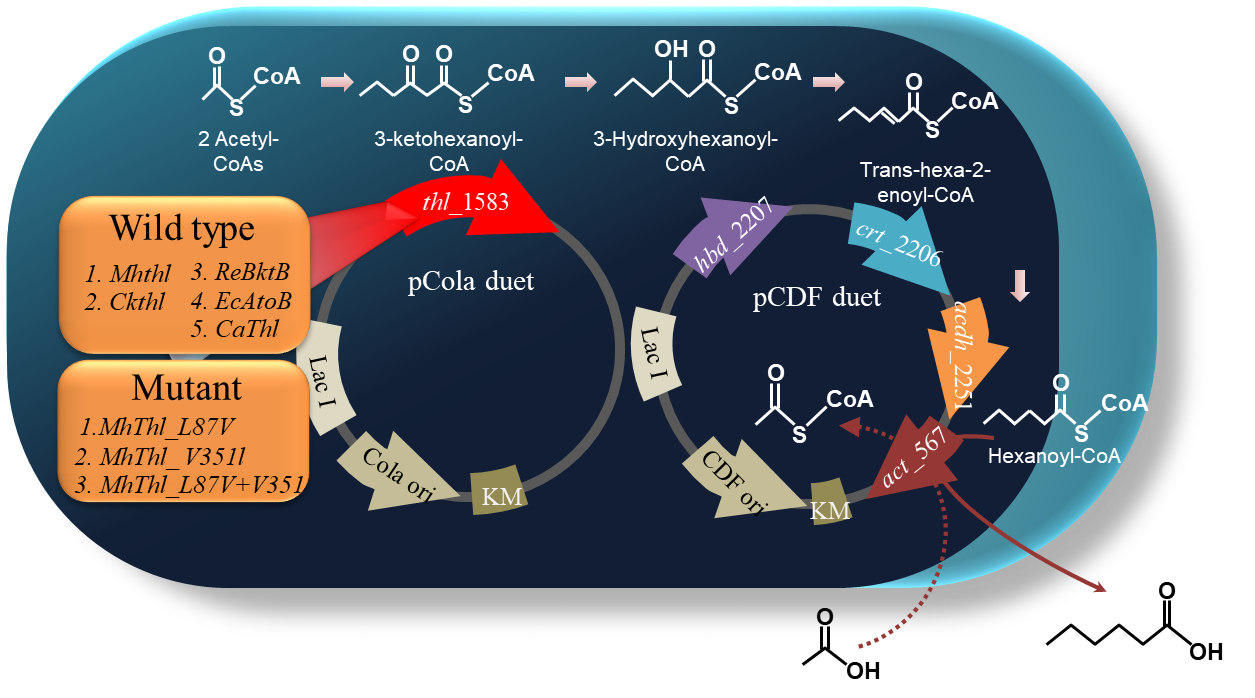


**Figure S13.** Modular *E. coli* platform strain to evaluate thiolase performance; the respective *thl* gene was co-expressed with *hbd*_2207, *crt*_2206, *acdh*_2251, and *act*_567.

**Table S10.** *Escherichia coli* strains used to construct metabolic pathways producing *n*-caproate and identify responsible genes for *n*-caproate production

| **Name** | | **Relevant characteristic** | **Source** |
| --- | --- | --- | --- |
| Strain | |  |  |
| DSM01 | | MG1655::λ(DE3)△frdA::FRT△pta::FRT△ldhA::FRT△adhE::FRT | Baek et al., 2013 |
| SK-1 | | DSM01/p*Mh*thl,pHC-AA | This study |
| SK-2 | | DSM01/p*Mh*thl, pHC-ATA | This study |
| SK-3 | | DSM01/p*Mh*thl, pHC-AETA | This study |
| SK-4 | | DSM01/p*Mh*thl, pHC-AEA | This study |
| SK-5 | | DSM01/p*Mh*thl, pHC-ABEA | This study |
| SK-6 | | DSM01/p*Mh*thl, pHC-BEA | This study |
| SK-7 | | DSM01/p*Mh*thl, pHC-TA | This study |
| SK-8 | | DSM01/p*Mh*thl, pHC-BA | This study |
| SK-9 | | DSM01/p*Mh*thl, pHC-A | This study |
| SK-10 | | DSM01/p*Ck*thl, pHC-AA | This study |
| SK-11 | | DSM01/p*Re*BktB, pHC-AA | This study |
| SK-12 | | DSM01/p*Ec*atoB, pHC-AA | This study |
| SK-13 | | DSM01/p*Ca*thl, pHC-AA | This study |
| SK-14 | | DSM01/p*Mh*thl-M1, pHC-AA | This study |
| SK-15 | | DSM01/p*Mh*thl-M2, pHC-AA | This study |
| SK-16 | | DSM01/p*Mh*thl-M3, pHC-AA | This study |
| Plasmid | |  |  |
| pCOLADuet | | Expression vector, Km^R^, ColA ori | Novagen |
| pCDFDuet | | Expression vector, Sm^R^, CDF13 ori | Novagen |
| pHC-AA | | CDF13 ori, Sm^R^, P_lac_::hbd-crt-P_lac_::acdh-P_lac_::act | This study |
| pHC-ATA | | CDF13 ori, Sm^R^, P_lac_::hbd-crt-P_lac_::acdh-ter-P_lac_::act | This study |
| pHC-AETA | | CDF13 ori, Sm^R^, P_lac_::hbd-crt-P_lac_::acdh- etfαβ-ter-P_lac_::act | This study |
| pHC-AEA | | CDF13 ori, Sm^R^, P_lac_::hbd-crt-P_lac_::acdh-etfαβ-P_lac_::act | This study |
| pHC-ABEA | | CDF13 ori, Sm^R^, P_lac_::hbd-crt-P_lac_::acdh-bcdh- etfαβ -P_lac_::act | This study |
| pHC-BEA | | CDF13 ori, Sm^R^, P_lac_::hbd-crt-P_lac_::bcdh-etfαβ-P_lac_::act | This study |
| pHC-TA | | CDF13 ori, Sm^R^, P_lac_::hbd-crt-P_lac_::ter-P_lac_::act | This study |
| pHC-BA | | CDF13 ori, Sm^R^, P_lac_::hbd-crt-P_lac_::bcdh-P_lac_::act | This study |
| pHC-A | | CDF13 ori, Sm^R^, P_lac_::hbd-crt-P_lac_::acdh | This study |
| pHC-ABE | | CDF13 ori, Sm^R^, P_lac_::hbd-crt-P_lac_::acdh-bcdh-etfαβ | This study |
| p*Mh*thl | | ColAori, Km^R^, P_lac_::thl from *Megasphaera* sp. MH | This study |
| p*Ck*thl | | ColAori, Km^R^, P_lac_::thl from *C. kluyveri* DSM555 | This study |
| p*Re*BktB | | ColAori, Km^R^, P_lac_::BktB from *R. eutropha* | This study |
| p*Ec*atoB | | ColAori, Km^R^, P_lac_::atoB from *E. coli* K12 | This study |
| p*Ca*thl | | ColAori, Km^R^, P_lac_::thl from *C. acetobutylicum* ATCC 824 | This study |
| p*Mh*thl-M1 | | ColAori, Km^R^, P_lac_::*Mh*thl single mutant L87V | This study |
| p*Mh*thl-M2 | | ColAori, Km^R^, P_lac_::*Mh*thl single mutant V351I | This study |
| p*Mh*thl-M3 | ColAori, Km^R^, P_lac_::*Mh*thl double mutant L87V+V351I | | This study |

**Table S11.** Five wild-type thiolases and three mutant thiolases with their respective corresponding critical residues

| **THLs** | **Residues 87** | **Residues 351** | |
| --- | --- | --- | --- |
| *Mh*THL | Leu 87 | | Val 351 |
| *Ck*THL | Leu 87 | | Val 352 |
| *Re*BktB | Leu 89 | | Ile 352 |
| *Ec*AtoB | Val 87 | | Ile 351 |
| *Ca*THL | Val 87 | | Ile 350 |
| *Mh*THL single mutant (L87V) | Val 87 | | Val 351 |
| *Mh*THLsingle mutant (V351I) | Leu 87 | | Ile 351 |
| *Mh*THL double mutant (L87V+V351I) | Val 87 | | Ile 351 |

**Table S12.** PCR primers used to construct the metabolic pathway producing *n*-caproate and identify critical residues related to the substrate pocket

| **Gene** | **Sequence (5′–3′)** |
| --- | --- |
| ***Mh. Thl*** | Forward: AAAAA**CATATG**AAAAATGTGGTTATTGTGTCC |
|  | Reverse: AAAAA**CTCGAG**TTACATCACACATTTTAATGGCAACTG |
| ***Mh. Hbd*** | Forward: AAAAA**GAATTC**GATGTTCAAGAAAGTGATGGTCATT |
|  | Reverse: AAAAA**CCCGGG**TTATTTAGAGTAATCGTAGAAGCCTT |
| ***Mh. Crt*** | Forward: AAAAA**cccggg**gcatgcaaggagatataggtaccccggtaccATGGAATTTGAAAACATCCTGTTCA |
|  | Reverse: AAAAA**CCGCGGCG** TTATTCGCCTTTGAAGTCCGC |
| ***Mh. Acdh*** | Forward: AAAAA**CTCGAG**ATGGGTTATATTCTTAACAAAGACCA |
|  | Reverse: AAAAA**CTCGAG**TTAGTGTTTCTTTGTAATCTGGCC |
| ***Td. Ter*** | Forward: AAAAA**CTCGAG**ATGATTGTTAAACCGATGGTCCG |
|  | Reverse: AAAAA**AAGCTT**TCAAATGCGATCAAAGCGTTCC |
| ***Mh. Bcdh*** | Forward: AAAAA**CATATG**GATATCTCTAGAATGGACTTCAAACTGAA |
|  | Reverse: AAAAA**CATATGgaattc**TCATTTCAGCAGGAAACCCGAC |
| ***Mh. etfAB*** | Forward:  Extension primer 1. AAAAA**GAATTC**ACTAGTCCTATCCTTGTTTATGAGGCA  Extension primer 2. GCCATTAGCGCTGTTGACATAAAAATTATTTTTAGGAGGCAACAGAAATGGGCCCATGGAAATCCTGGTGTGCGTC |
|  | Reverse: AAAAA**aagctt**TCAGCCTTTGATCTTTTTGATGGCAC |
| ***Mh. act*** | Forward:  Extension primer 1. AAAAA **AAGCTT**CCGCGGGAGCTCTAATACGACTCACTATAGGGGAATTGTGAGCGGATAACAATTCCCCATCTTAGTAT  Extension primer 2. CGGATAACAATTCCCCATCTTAGTATATTAGTTAAGTATAAGAAGGAGATATAGTCGACGGATCCATGTATAAACTGTCGCAAATCGCT |
|  | Reverse: AAAAA**CTCGAG**TTAGTATTCCGTTTTTGAGGTTTTCGT |
| ***Ec. AtoB*** | Forward: Aaaaa**CATATG**AAAAACTGTGTTATTGTGTC |
|  | Reverse: AAAA**CTCGAG**TTAGAATTCATTCAGGCGTTCGATC |
| ***Ca. thl*** | Forward: AAAAAA**CATATG**CGTGATGTCGTGATTGT |
|  | Reverse: AAAAA**CTCGAG**TTAGAATTCATCACGTTCCACGAC |
| ***Re. BktB*** | Forward: AAAAA**CATATG**ACCCGTGAAGTTGTCGT |
|  | Reverse: AAAAA**CTCGAG**TTAGAATTCGATGCGTTCGAAGATGG |
| ***Ck. Thl*** | Forward: AAAAA**CATATG**CGTGAAGTGGTGATTGTGA |
|  | Reverse: AAAAA**CTCGAG**TCAACGTTCCACCACCACTG |
| ***Mh. thl_L87V (CTG-GTG)*** | Forward: GCGTTTACCATTAATAAA**GTG**CGGTTCAGGTCTG |
|  | Reverse: CAGACCTGAACCGCA**CAC**TTTATTAATGGTAAACGC |
| ***Mh. thl_V351I (GTC-ATC)*** | Forward: CTGGGTCACCCG**ATC**GGTTGTTCTGGTGCACGT |
|  | Reverse: ACGTGCACCAGAACAACC**GAT**CGGGTGACCCAG |


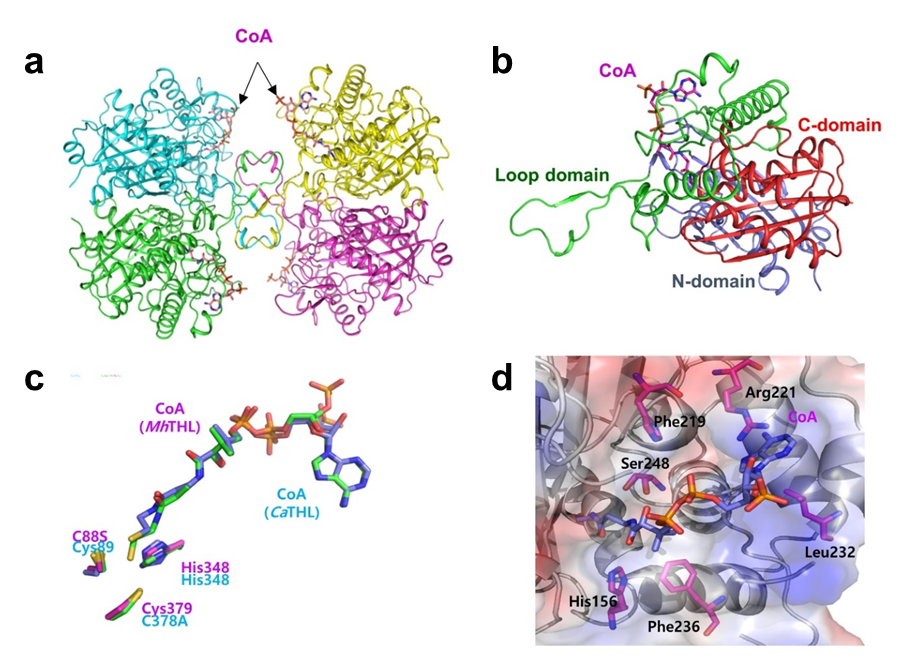


**Figure S14.** Overall structure and critical residues of the *Mh*THL **a** Overall shape of the *Mh*THL tetramer. Four polypeptides: cyan, green, yellow, and magenta. A stick model in magenta shows CoA molecules bound to each of the four polypeptides. **b** Cartoon diagram of the overall shape of the *Mh*THL monomer; light-blue: N-terminal, red: C-terminal, and green: Loop domain. A stick model in magenta shows the bound CoA. **c** Different CoA positions in *Mh*THL and *Ca*THL. CoA-bound forms of *Mh*THL and *Ca*THL are superposed, and the bound-CoAs are shown with stick models. Three catalytic residues of *Mh*THL and *Ca*THL are shown with stick models. **d** Substrate binding pocket of *Mh*THL. *Mh*THL structure is presented as a cartoon and electrostatic surface potential model. Residues involved in the substrate binding are shown as a stick model in orange. Bound CoA is shown as a stick model in magenta.


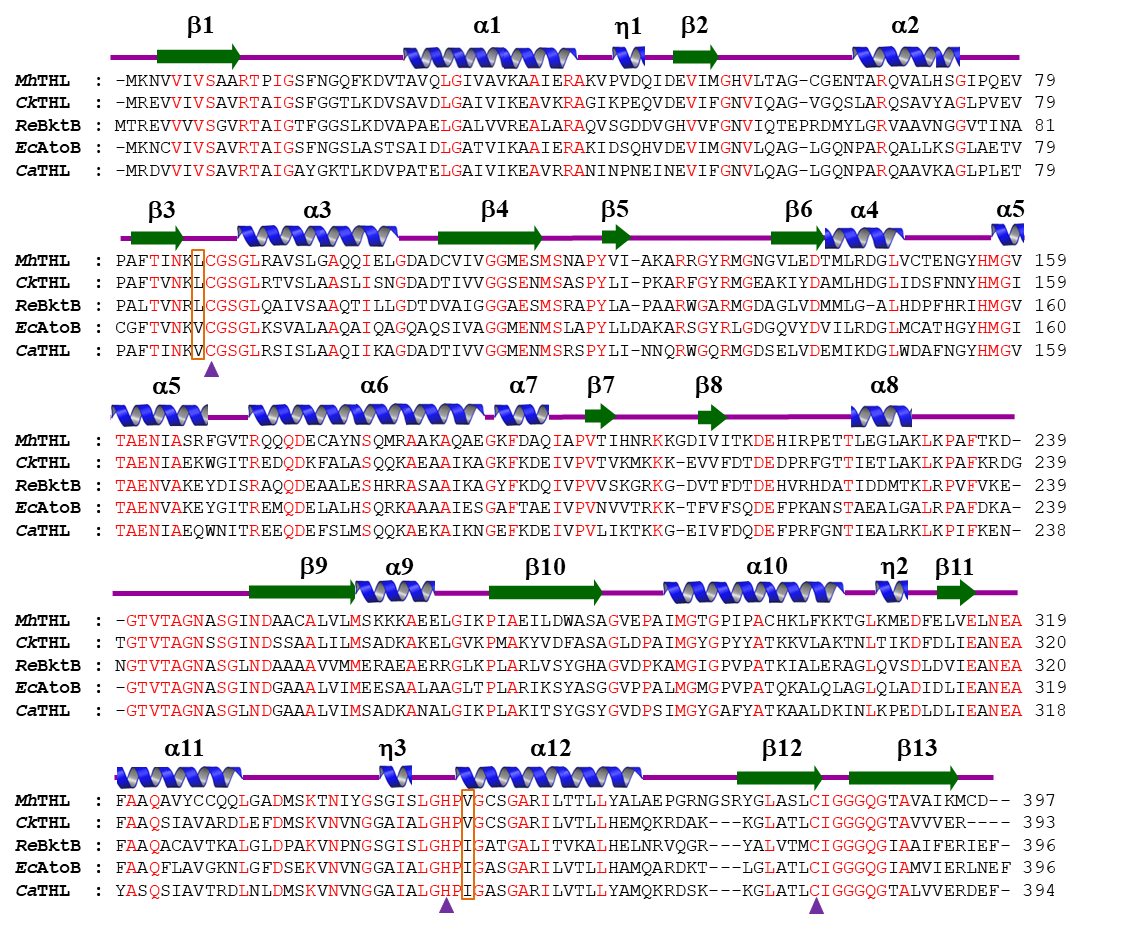


**Figure S15**. Amino acid sequence alignment of thiolases. Secondary structure of *Mh*THL is shown and labeled. Identical amino acid sequences are distinguished with red color. Three purple triangles and two orange boxes indicate catalytic residues and positions of the key residues for utilizing acyl-CoA molecules as acyl group donors, respectively.


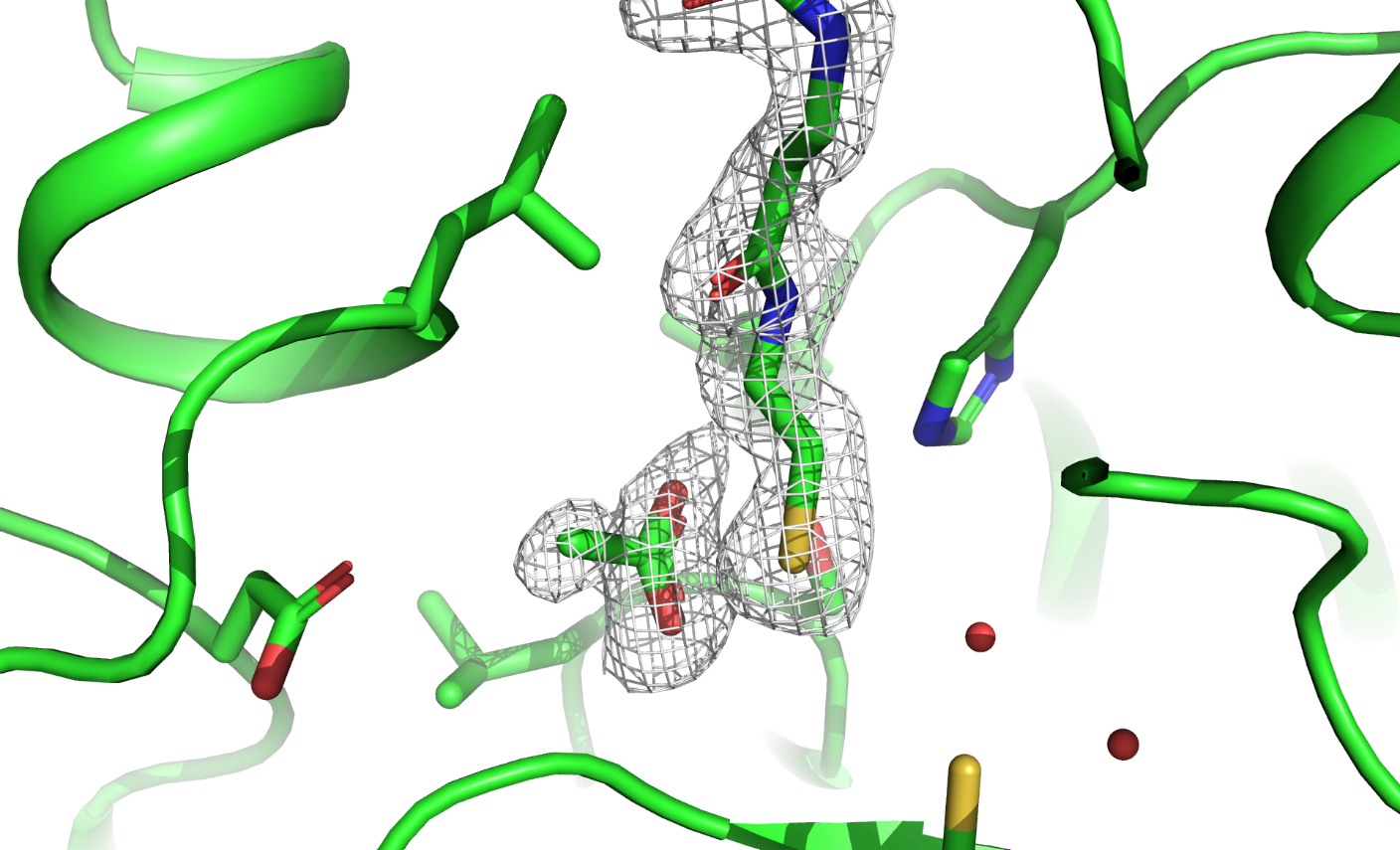


**Acetate**

**CoA**

**H2O**

L148

V351

L87

E63

C88S

H349

C382

**Figure S16.** Fo–Fc omit map for CoA and acetate molecules. The electron density map is shown with gray meshes contoured at 2.0 Å. The *Mh*THL structure is shown as a green cartoon, and residues in the environment are shown with stick models.


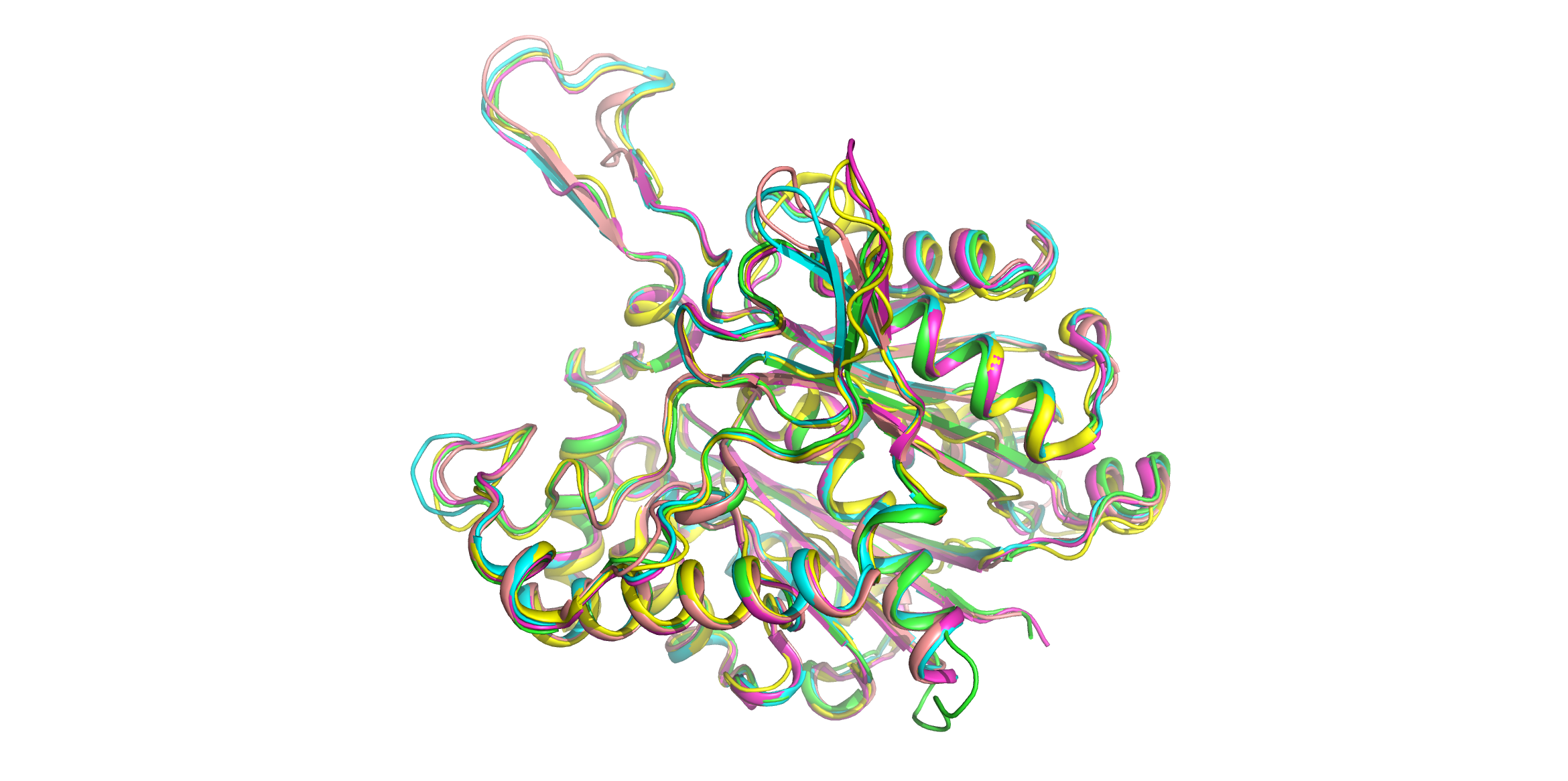


***ReBktB***

***EcAtoB***

***CaTHL***

***CkTHL(Simulated)***

***MhTHL***

**Figure S17.** Overall structure superposition. The crystal structures of *Ec*AtoB (Salmon), *Re*BktB (Yellow), *Ca*THL (Magenta), *Mh*THL (Green), and *Ck*THL (Cyan) are shown.


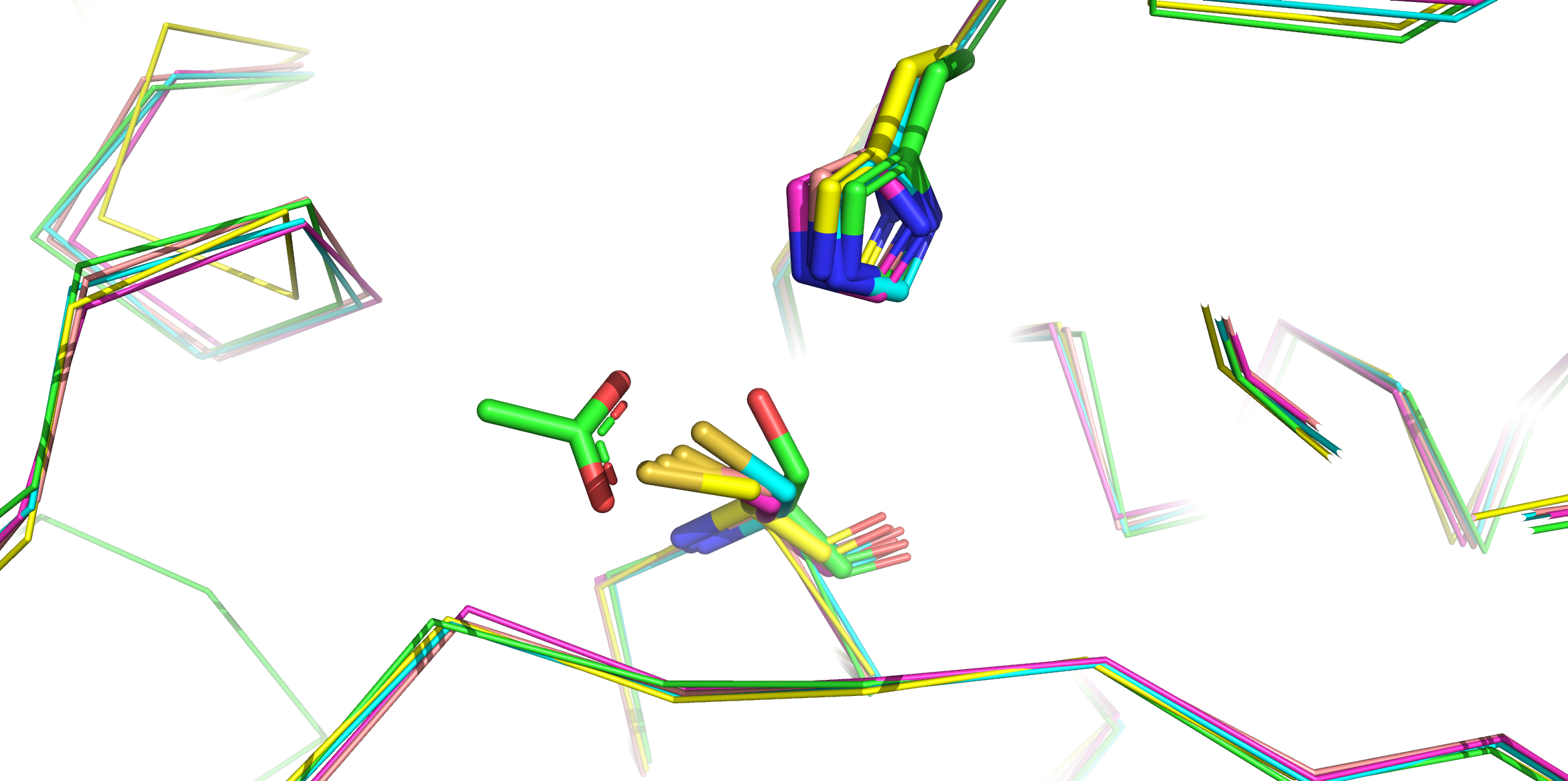


***ReBktB***

***EcAtoB***

***CaTHL***

***MhTHL***

***CkTHL***

(Simulated)

**Oγ**

**Sγ**

**Sγ**

**Sγ**

**Sγ**

**Cβ**

**Cα**

**N**

**Sγ(Oγ)-Cβ-Cα-N Dihed.**

***MhTHL***

*EcAtoB*

*ReBktB*

*CaTHL*

*CkTHL*

**-69.6°**

-51.8°

-42.2°

-27.4°

-28.4°

*simulated*

-36.9°

(avg.)

**Figure S18.** Oγ-Cβ-Cα-N dihedral angles of C88S in *Mh*THL and corresponding cysteine residues in other structures The crystal structures of *Ec*AtoB (Salmon), *Re*BktB (Yellow), *Ca*THL (Magenta), *Mh*THL (Green), and *Ck*THL (Cyan) are shown in a ribbon model. C88S and its corresponding residues in the other thiolases are shown as a stick model. The table shows the dihedral angle values.

**Table S13**. Data collection and refinement of statistics

|  | ***Mh*THL** |
| --- | --- |
|  | **C88S variant, soaked with hexanoyl-coenzyme A** |
| **Data collection** |  |
| Space group | *P*22_1_2_1_ |
| Cell dimensions |  |
| *a*, *b*, *c* (Å) | 51.505, 111.443, 140.996 |
| a, b, g (°) | 90.00, 90.00, 90.00 |
| Resolution (Å) | 38.99-1.64 (1.70–1.64) |
| Total reflections | 199403 (19630) |
| Unique reflections | 99877 (9827) |
| *R*_sym_ or *R*_merge_ | 2.374 (27.68) |
| CC1/2 | 0.999 (0.853) |
| *I*/s*I* | 12.08 (2.11) |
| Completeness (%) | 99.6 (99.2) |
| Redundancy | 2.0 (2.0) |
|  |  |
| **Refinement** |  |
| Resolution (Å) | 38.99–1.64 |
| No. reflections | 92490 |
| *R*_work_/*R*_free_ | 19.82/23.56 |
| Average B-factor | 25.0 |
| No. atoms | 12017 |
| Protein | 5872 |
| Water | 624 |
| RMS deviation |  |
| Bond lengths (Å) | 0.008 |
| Bond angles (°) | 1.549 |
| Ramachandran plot |  |
| Favoured (%) | 94.9 |
| Outliers (%) | 1.18 |

**Supplementary References**

[1] a) Y. Dekishima, E. I. Lan, C. R. Shen, K. M. Cho, J. C. Liao, J Am Chem Soc 2011, 133 (30), 11399, https://doi.org/10.1021/ja203814d ; b) H. B. Machado, Y. Dekishima, H. Luo, E. I. Lan, J. C. Liao, Metab Eng 2012, 14 (5), 504, https://doi.org/10.1016/j.ymben.2012.07.002.

[2] A. R. Volker, D. S. Gogerty, C. Bartholomay, T. Hennen-Bierwagen, H. Zhu, T. A. Bobik, Microbiology (Reading) 2014, 160 (Pt 7), 1513, https://doi.org/10.1099/mic.0.078329-0.

[3] E. J. Kim, H. F. Son, S. Kim, J. W. Ahn, K. J. Kim, Biochem Bioph Res Co 2014, 444 (3), 365, https://doi.org/10.1016/j.bbrc.2014.01.055.

[4] J. H. Lim, S. W. Seo, S. Y. Kim, G. Y. Jung, Bioresour Technol 2013, 135, 568, https://doi.org/10.1016/j.biortech.2012.09.091.

[5] M. Yu, Y. Zhang, I. C. Tang, S. T. Yang, Metab Eng 2011, 13 (4), 373, https://doi.org/10.1016/j.ymben.2011.04.002.
